# OBIMAP (One-Bead Interchain Multipeptide Assembly Platform)

**DOI:** 10.1021/acsbiomedchemau.5c00237

**Published:** 2026-01-14

**Authors:** Othman Al Musaimi, Daryl R. Williams

**Affiliations:** † School of Pharmacy, 12186 Newcastle University, Newcastle upon Tyne NE1 7RU, U.K.; ‡ Translational and Clinical Research Institute, Faculty of Medical Sciences, Newcastle University, Newcastle Upon Tyne NE1 7RU, U.K.; § Department of Chemical Engineering, Imperial College London, London SW7 2AZ, U.K.; ∥ Orthogonal Peptides Limited, London SW7 2AZ, U.K.

**Keywords:** solid-phase peptide synthesis, interchain assembly, cyclic peptides, bicyclic peptides, constrained peptides, cross-linked peptides, therapeutic peptides

## Abstract

A significant advancement in Merrifield’s classic solid-phase peptide synthesis (SPPS) that greatly expands the scope of accessible peptide structures is reported here. Building upon the one-bead, one-compound (OBOC) concept, this approach enables the simultaneous synthesis of multiple peptides on a single bead, followed by a novel solid-phase interchain assembly reaction to produce the final peptide product. This method, the one-bead interchain multipeptide assembly platform (OBIMAP), successfully generates diverse peptide architectures, including linear, cyclic, and bicyclic structures—ranging from minimal cyclic dipeptides to small proteins—many of which are inaccessible through conventional SPPS. OBIMAP demonstrates superior efficiency in both time and product purity compared to traditional methods. Crucially, it eliminates the need for solution-phase fragment condensation, a common but cumbersome step commonly used in synthesizing therapeutic peptides (30–60 amino acids). In addition to enhancing conventional SPPS methodologies, the OBIMAP enables access to novel classes of peptide architectures, including highly constrained peptides that were previously considered synthetically inaccessible.

## Graphical abstract

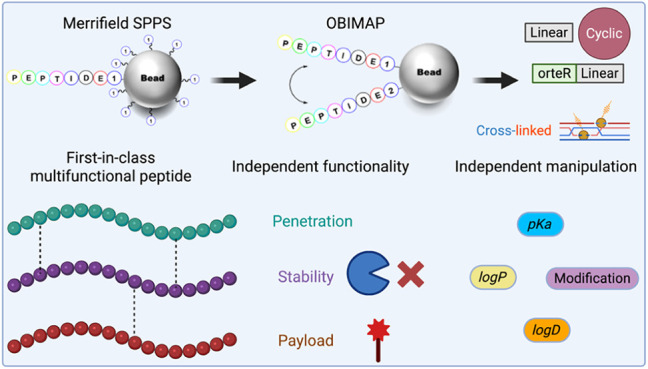

## 1 Introduction

Peptides can be sourced from nature, chemical synthesis, or library screening, with phage display and advanced systems like RaPID enabling rapid discovery.^1–3^ Key synthesis methods include classical solution-phase peptide synthesis (CSPS), solid-phase peptide synthesis (SPPS), liquid-phase peptide synthesis (LPPS), and native chemical ligation (NCL), the latter is accelerated by α-selenoesters for efficient long-peptide assembly.^4–6^ Innovations such as mRNA display, split-intein ligation, and genome mining with BGC analysis (e.g., AntiSMASH, DeepRiPP) have uncovered diverse RiPPs, including lasso peptides with rare post-translational modifications (PTMs) that enhance stability, permeability, and specificity.^7–9^ Naturally derived peptides show antimicrobial, anticancer, and immunomodulatory activity; brentuximab vedotin, an FDA-approved ADC, exemplifies their clinical potential.^10–13^ Structural modifications to charge, hydrophobicity, and helicity further improve targeting, as cationic peptides selectively bind negatively charged membranes.^10,14^

SPPS was introduced in 1963 by Nobel Laureate Merrifield.^15^ In the standard SPPS methodology, a specific peptide is grown on the solid support particles one amino acid at a time, with a multitude of identical peptide chains being assembled on each bead of a polymeric solid support resin.^15^ The synthesis is a repetitive process that includes sequential deprotection, coupling, and washing reaction steps to elongate these chains until the desired single peptide is assembled. These identical peptide chains are all elongated simultaneously to produce the identical peptide in a single overall process. At the end of the process, the peptide is chemically cleaved from the resin with the aid of the appropriate reagents. The Merrifield protocol is a straightforward and conventional method used in the SPPS process.^15^ Since its invention, tremendous efforts have been invested to advance this technology including a range of new solid supports,^16–20^ new coupling reagents,^21^ as well as greener reaction technologies to meet environmental legal authorities’ requirements, thus protecting humankind and the environment.^22–24^ However, its core protocol has remained virtually unchanged for the past 60 years.

Despite its success at both research and industrial scales, conventional SPPS often requires that some key reactions be performed off the resin.^25,26^ This requirement is primarily due to undesired side reactions occurring, restricting current SPPS practice to typically 15 to 20 amino-acid-long peptides, where purity and yield can become problematic commercially.^25^ Therefore, common current commercial practice for manufacturing a peptide of say 45 amino acids is to synthesize three 15 amino-acid peptide fragments, and sometimes shorter fragments, then combine these fragments in a solution condensation reaction.^25^ This approach requires detaching each peptide precursor from the resin (once for each fragment, then purifying each as necessary) and then carrying out the subsequent assembly reaction. These additional steps impose a significant penalty on the yield and purity of the product, as well as the environmental footprint of the process.^27^

Panigrahi and colleagues have developed a small-molecule catalyst for peptide synthesis that employs redox recycling of diselenide and phosphine, using air as the terminal oxidant and phenylsilane as the reductant.^28^ The catalyst enables efficient amino acid coupling in both solution and solid phase and is compatible with acetonitrile, eliminating the need for DMF.^28^ However, this approach is limited to resins that swell in acetonitrile, such as Tentagel, and is not applicable to the widely used polystyrene-based resins.^22,28^ Bürgisser et al. developed a “Synthesis Tag” (SynTag), consisting of six arginines linked to the target sequence via a cleavable MeDbz linker, which facilitates batch- and flow-SPPS of difficult sequences, improves peptide solubility, and enables direct access to native sequences by hydrolysis or peptide thioesters for NCL.^29^ Hattori and Yamamoto have developed a method using five-membered rings formed from unprotected amino acids with Si or Al reagents, which react with amino acid *tert*-butyl esters.^30,31^ Despite these advances, peptide bond formation between fully unprotected amino acids remains challenging due to side reactions such as self-condensation, inversion, cyclization, and overreaction.^32^ The same group later reported the formation of silacyclic dipeptides via cross-condensation between two unprotected amino acids—the first such example—yielding stable silacyclic intermediates that can be efficiently elongated at both *C*- and *N*-termini without coupling reagents.^32^

Here, the reinvention of the standard SPPS process is proposed in two new and novel ways. First, multiple different peptides are concurrently designed and synthesized in the SPPS on a single bead. The second novel aspect of this work is a new interchain assembly reaction, which allows to synthesize a wide spectrum of valuable peptide therapeutics by chemically driving the two or three precursor peptide chains synthesized on the bead to react with each other to form the final peptide product (Figure 1).

**Figure 1:**
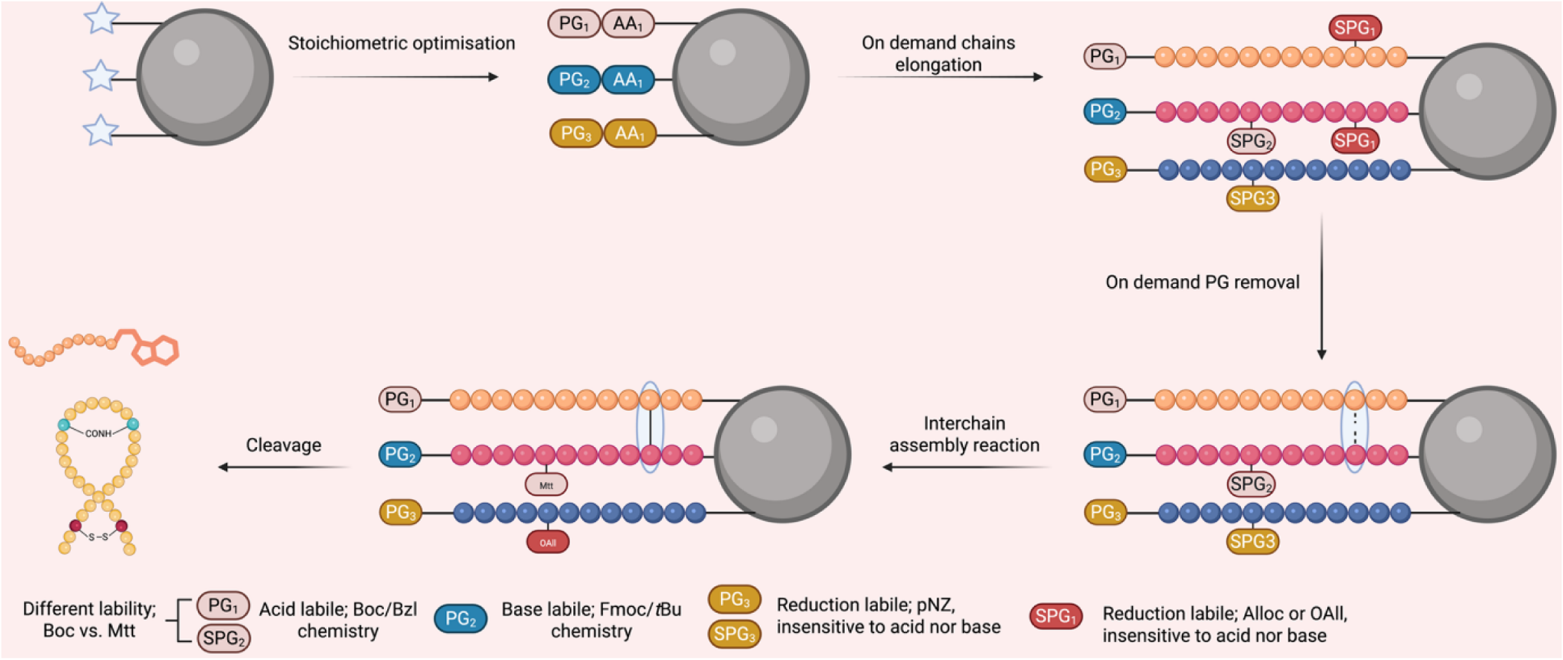
Schematic representation of the OBIMAP technology. Boc, *tert*-butoxycarbonyl; Bzl, benzyl; Fmoc, 9-fluorenylmethoxycarbonyl, pNZ; *p*-nitrobenzyloxycarbonyl, Mtt; 4-Methyltrityl.

The first part of the current innovation is derived from an approach currently used to synthesize a diverse range of peptides with potential therapeutic applications, the one-bead, one-compound (OBOC) approach, which has been known since 1991.^33^ It is an efficient strategy that utilizes the SPPS approach but prepares a huge library of peptides, thousands and even millions of different peptides. Afterward, this peptide library is mixed with a target molecule, and the functional peptide can be identified accordingly, which binds to the target molecule. Although inspired by the OBOC paradigm, the OBIMAP is fundamentally distinct. OBOC employs SPPS to generate random peptide libraries, from which functional sequences are identified through target binding.^33^ In contrast, OBIMAP relies on the independent, rational design and synthesis of peptide chains, ensuring a full sequence definition of each assembled component. A central innovation is the on-resin interchain assembly reaction used to combine peptide chains, enabling the production of both established industrial peptides and first-in-class therapeutic candidates. This capability is absent in OBOC methodologies, which do not incorporate preplanned protection or assembly strategies. Moreover, whereas OBOC libraries are typically limited to short peptides (up to four amino acids),^33^ OBIMAP supports the synthesis of peptides of up to 60 amino acids in length.

The combination of these two innovations expands the synthesis landscape of existing peptides, as well as allowing the creation of first-in-class peptide families with potential therapeutic applications in an easier, faster, and more cost-effective reaction process. For instance, in OBIMAP, purification will only be required once at the end of the synthesis. Therefore, in comparison to the solution-phase coupling, OBIMAP uses fewer steps and hence achieves higher yields. Most importantly, OBIMAP fully inhibits dimerization reactions by having one of the termini anchored to the resin during the course of the reaction.^34^ In conclusion, OBIMAP is a major advance in SPPS and entirely replaces the current hybrid method of using SPPS to produce the precursor peptide chains, which are in turn reacted in solution to create the final target larger peptide products (Table 1).^34^

**Table 1:** Comparison between OBIMAP and the Existing SPPS Approach

Existing SPPS technology^15^	OBIMAP technology	Impact
In solution, oligomerization side reaction is most likely to occur.	Oligomerization is entirely inhibited (*C*-terminal is anchored to the resin).	Purer product. Higher yield.
Requires ∼3× excess of reacting species.	Reduced stoichiometries (by a factor of 3).	Reduced cost and waste.
Multiple purifications are required.	One purification step might be needed at the end.	Higher yield. Reduced cost, waste, time ,and effort.
Can synthesize up to 20- or 30-mer peptides purely in solid settings.	Can synthesize more than 60-mer peptides purely in solid settings.	Reactions in solution are completely omitted. Reduced cost, waste, time, and effort.
Solubility of peptide fragments is prerequisite, and it could be detrimental. Aggregation of fragment solution is a dilemma.	Solubility of peptide fragments is not prerequisite as they are anchored onto the resin.	Versatility to synthesize any peptide with any amino acid composition.
Can only synthesize common peptide structures.	Can synthesize common and novel peptide structures (e.g., cross-linked).	Wide range of new potential therapeutic and industrial peptides.

## 2 Results and Discussion

The process for preparing a peptide or peptide-like compound involves: (i) providing a first peptide fragment bearing a first reactive functional group, (ii) providing a second peptide fragment bearing a second reactive functional group, and (iii) coupling the first and second reactive groups to form a covalent bond. The terms “first reactive functional group” and “second reactive functional group” refer to functional groups that are capable, under suitable conditions, of reacting with one another to form a covalent bond. All peptide fragments are synthesized on a polymer support using standard stepwise SPPS. An *N*-α-protected, if necessary, side-chain—with protected *C*-terminal amino acid—is coupled to the swollen resin directly or via a linker, followed by iterative *N*-α deprotection and amino acid coupling to extend the chain.^35^ Upon completion, side-chain deprotection and resin cleavage are performed either sequentially or concurrently.^20^

Any established peptide protection strategy can be employed. Preferred approaches utilize either Boc or Fmoc *N*-α protection with compatible side chain protecting groups. An additional orthogonal protecting group may also be incorporated, preferably p-Nitrobenzyloxycarbonyl (pNZ), which is orthogonal to both Boc and Fmoc.^36^ A nonlimiting selection of solid supports suitable for the preparation of peptide amides includes Merrifield polystyrene-based resin, PAM resin, Wang resin, Rink amide resin, PAL resin, Sieber amide resin, MBHA resin, and trityl or 2-chlorotrityl resins. Base-labile supports may also be employed, including oxime resin, HMBA resin, DHP resin, Weinreb aminomethyl resin, and polyethylene glycol–polystyrene grafted resins. Details of these resins are well documented in the literature.^37^ Suitable reaction conditions are well established; for example, amide bonds can be formed via amine–carboxylic acid coupling using standard reagents (e.g., carbodiimides, phosphonium salts), ester bonds via alcohol–carboxylate coupling, disulfide bonds under oxidizing conditions, and C–C bonds using established metathesis-based stapling strategies.^38,39^ In addition to direct coupling between the first and second reactive functional groups, bond formation may also occur via an intervening linker, which refers to a chemical moiety capable of reacting with both reactive groups, typically as a bivalent entity. Representative linkers include dicarboxylic acids (e.g., oxalic, malonic, succinic, diglycolic, glutaric, adipic, pimelic, and suberic acids), diols, diamines, and amino acids.^40^ Nonlimiting examples of such reactive group pairs and the corresponding bonds formed are provided in Table 2.

**Table 2:** Nonexhaustive Examples of Corresponding First and Second Reactive Groups and the Resulting Bonds Formed

First reactive group (A)	Second reactive group (B)	Bond formed
Amine (−NHR; R = H, Alkyl)	Carboxyl group (−COX; X = halogen, OH, OAlkyl)	Amide −NHRCO–
Alcohol (−OH)	Carboxyl group (−COX; X = halogen, OH, OAlkyl)	Ester −OCO–
Thiol (−SH)	Thiol (−SH)	Disulfide
Alkene	Alkene	C–C bond
Alcohol (−OH)	Alcohol (−OH)	Ester
Azide (−N_3_)	Alkyne (−C≡C−)	Triazole

### 2.1 Core Concept

Products (1) and (2) are formed via side-chain-to-side-chain coupling, including reactions between the reactive functional group A (ε-amine of lysine or the hydroxyl group of serine) and the reactive functional group B (carboxylic acid side chain of aspartic or glutamic acid, or thiol–thiol bridging between cysteine residues) from separate chains (Figure 2).

**Figure 2:**
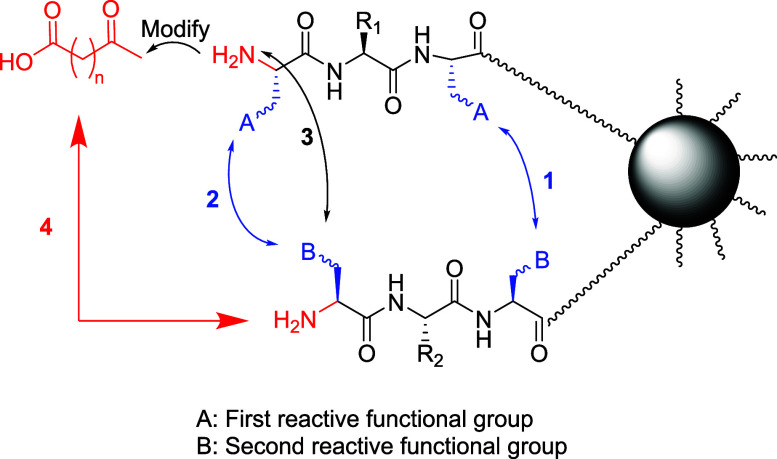
Chemistry of peptide chains assembled and linked on one bead via the OBIMAP.

Product (3) is generated through a head-to-side-chain linkage by coupling the *N*-terminus of one chain to the reactive functional group (carboxylate side chain of aspartic or glutamic acid) on the partner chain. Product (4) is obtained via head-to-head coupling of two *N*-termini following installation of an electrophilic functionality as a chemical moiety on one chain, preserving carboxylate-based chemistries. The first three assembly strategies require strategically positioned amino acids (Asp, Glu, Lys, Orn, Ser, Tyr, or Cys) to serve as conjugation sites, whereas the head-to-head approach is sequence-agnostic and applicable to any *N*-terminal residue with a free amino functionality.

### 2.2 Proof of Concept

By employing orthogonal protecting strategies, specialized linkers, and selective chemical groups, the simultaneous synthesis of two or three distinct peptide chains on a single bead, rather than just one, is facilitated. The process begins by elongating a resin-anchored peptide chain to the desired length. Next, a mixture of two differentially protected amino acids—Fmoc-Asp­(OAll)–OH and Fmoc-Lys­(Alloc)–OH—was coupled to the anchored chain, generating two separate peptides differing only in their *N*-terminal residue (Asp-peptide and Lys-peptide). The allyl (All) protecting group was strategically selected because it can be selectively cleaved from the side chains of both Asp and Lys, while the peptides remain attached to the resin. This orthogonal deprotection approach facilitated an interchain assembly reaction between the newly exposed carboxylic acid (Asp) and amine (Lys) groups, enabling further structural diversification.

The following sections present the variety of peptide structures accessible through OBIMAP, including linear peptides with a 2-fold increase in terminal chain assembly, along with ultrashort cyclic, cyclic, bicyclic, partially modified retro (PMR), and cross-linked peptides (Figure 3).

**Figure 3:**
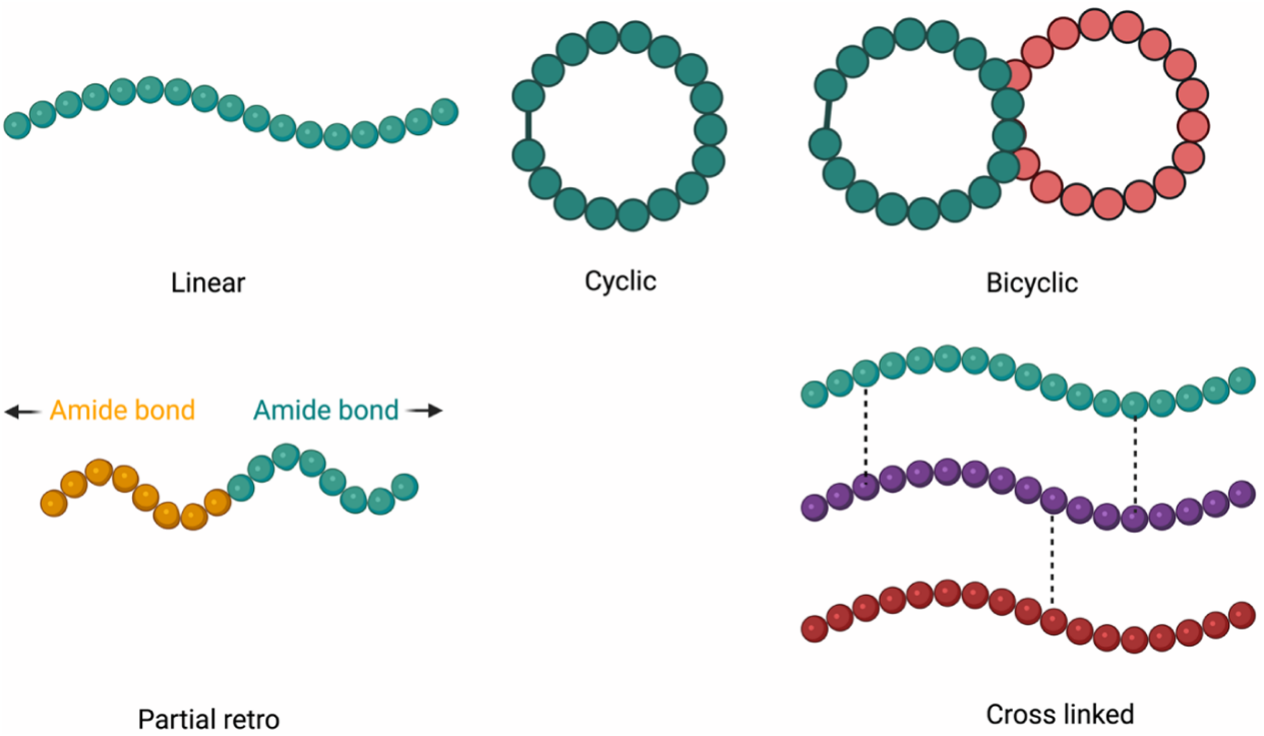
Chemical structures of peptides are accessed via the OBIMAP.

### 2.3 Linear Peptide Synthesis

OBIMAP enables the construction of novel linear peptides through interchain assembly of immobilized peptide chains. Unlike conventional linear peptides, which possess only *N*- and *C*-termini, the resulting assemblies contain multiple termini proportional to the number of fused chains. These termini provide orthogonal handles for the attachment of targeting moieties, thereby enhancing the receptor-binding potential.

An octapeptide was prepared by ligating two peptide segments. The sequences of these segments were identical except for a point variation, with one featuring an Asp residue and the other a Lys residue at the equivalent position (Figure S1). 2-Chlorotrityl chloride (CTC) was used in this synthesis. The mass of the peptide was confirmed by MS (Figures S2 and S3).

This work has demonstrated the key success of this new concept. Now, the resin was loaded with equimolar amounts of each reacting species, i.e., Lys and Asp. An excess of unreacted Asp-containing precursor (Fmoc-GDGL–OH) was observed and confirmed by MS (Figure S4). Thus, a decision was made to optimize this reaction and determine the optimum loading percentages of each of these two reacting species so as to obtain equimolar numbers of both sites on the resin.

### 2.4 Procedure Optimization

Reaction stoichiometry is critical to the success of this novel synthetic approach. Incomplete consumption of the reacting species compromises the process: a deficiency of one amino acid terminates the primary reaction pathway, whereas an excess promotes side reactions that reduce the product purity and yield and may ultimately lead to complete synthesis failure.

Initial experiments, guided by observations from linear peptide synthesis, involved the reaction of Asp with Lys-containing chains on a preloaded Gly-CTC resin in an equimolar (1:1) ratio. Under these conditions, unequal reactivity was observed, as indicated by a persistent excess of one reactant. Analysis of the interchain assembly reaction revealed an accumulation of unreacted Asp, which corroborated these findings. The yield of the target peptide relative to the remaining Asp was limited to 85% (Figure S5), demonstrating that true equimolar incorporation was not achieved. This behavior can be rationalized by differences in *C*-terminal reactivity, which likely result in preferential incorporation of one component over the other. Consequently, a range of stoichiometric ratios were investigated to enable complete consumption of both chains.

To drive the reaction to completion and minimize side-product formation, the loading ratio was adjusted to reduce the amount of unreacted Asp. A loading ratio of 33% Asp to 67% Lys was found to provide the required effective equimolar stoichiometry. This optimization led to near-complete consumption of Asp, increased the final peptide yield to >95% (Figure S6), and improved product purity by suppressing competing side reactions. Accordingly, this optimized ratio of 33% Asp to 67% Lys was adopted for all subsequent studies reported herein.

### 2.5 Cyclic Peptide Analogues

Cyclic peptides exhibit superior metabolic stability due to their conformational rigidity and are powerful tools for cell penetration.^41^ These properties, combined with their ability to engage extended protein–protein interaction (PPI) surfaces, make them ideal therapeutic candidates.^42^ Peptide cyclization enhances stability by reducing enzymatic degradation pathways and restricting conformational flexibility.^43^ Head-to-tail cyclization masks terminal groups, while side-to-side (e.g., disulfide bonds in insulin) and head-to-side linkages further improve proteolysis resistance. Peptide stapling similarly stabilizes α-helices, boosting membrane permeability.^44,45^ Disulfide bridges stabilize secondary structures including turns, helices, and sheets,^46–48^ also shield peptides from proteases, and enhance target binding.^49^ They also lower surface polarity, improving permeability, while allowing controlled biodegradation via thiol-mediated reduction.^50^ This dual stability-clearance property makes disulfide cyclization especially valuable for drug design.^51^

Nielsen et al. studied 125 cyclic peptides (4–37 residues) absorbed in mammals, showing that cyclization improves metabolic stability and bioactive conformations by masking termini and modulating polar group exposure.^50^ However, most peptides still showed poor oral bioavailability, with only a few advancing to preclinical or clinical evaluation.^50^

Building upon the successful synthesis of the linear peptide analogue, we next targeted a cyclic derivative to exploit the advantages of a constrained structure. OBIMAP enables complete solid-phase delivery of cyclic peptides, eliminating the need for solution-phase steps and advancing conventional SPPS. To achieve cyclization, the two previously optimized chains were elongated with additional Gly residues, followed by the incorporation of the orthogonally protected Asp­(OAll) and Lys­(Alloc) amino acids to introduce a second junction point. The interchain assembly protocol established for the linear analogue was then applied, successfully forming the cyclic peptide via a side-chain-to-side-chain bond (Figure S7). The identity of the macrocycle was confirmed by MS (Figures S8 and S9).

To evaluate the general applicability of our cyclization strategy, we investigated different solid supports. While the initial synthesis utilized CTC resin, we successfully prepared the cyclic peptide analogue (Figure S10) on Rink amide resin. The identity of the product was confirmed by MS (Figures S11 and S12), demonstrating the robustness of this approach across different solid supports.

### 2.6 Bicyclic Peptide

The versatility of this synthesis protocol enables the creation of diverse architectures by linking two peptide chains at multiple sites. To demonstrate this, we applied the method to prepare a bicyclic peptide—a class of molecules that is typically difficult to access and often requires solution-phase synthesis. A bicyclic structure was successfully constructed by forming three distinct linkages between the two chains (Figure 4). The mass of the product was confirmed by MS (Figures S13 and S14).

**Figure 4:**
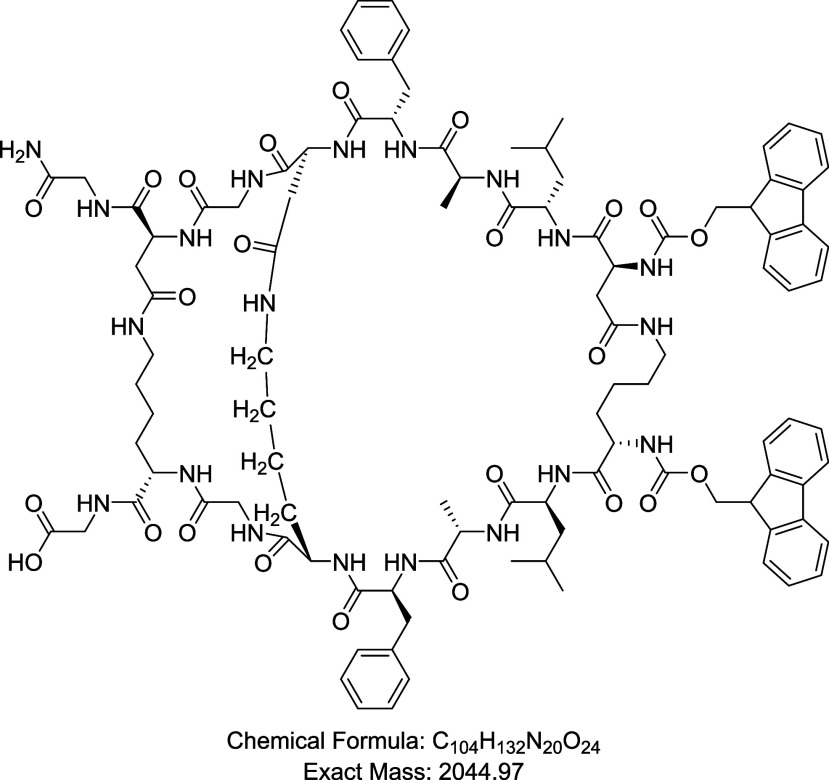
Chemical structure of a bicyclic peptide on a Rink amide resin.

### 2.7 Highly Constrained Analogues

The cyclization of short peptides (fewer than 7 amino acids) is notoriously difficult due to high steric hindrance and an inability to bend/distort into the necessary conformation. Traditional methods, whether in solution or on solid support, frequently result in undesirable byproducts like cyclodimers and epimerized compounds.^46^ OBIMAP, however, demonstrated its capability by enabling the successful synthesis of a highly challenging cyclic peptide, as shown in Figure S15.

The synthesis of this challenging cyclic peptide was successful, and its identity was confirmed by MS (Figures S16 and S17). It is noteworthy that the high conformational constraint of the structure necessitated an extended reaction time of 48 h—double the standard 24 h—to achieve complete conversion. Furthermore, it was demonstrated that OBIMAP is not limited to canonical amino acids. The methodology was successfully applied to incorporate the unnatural amino acid ornithine (Orn), which features a side chain and one methylene group shorter than that of lysine. An Orn-containing peptide was efficiently reacted with an Asp-containing chain to yield the final product, confirming the protocol’s versatility for residues with shorter side chains (data not shown).

### 2.8 Partially Modified Retro Peptide (PMR)

The retro-inverso (RI) concept, pioneered by Professor Goodman in the 1970s, has been widely adopted in fields ranging from immunology to drug discovery.^52,53^ Partially modified retro inverso (PMRI) peptides, in particular, offer a promising strategy to enhance the metabolic stability of biologically active peptides—a critical hurdle in their therapeutic application.^54^ However, current synthetic routes for PMRI peptides necessitate additional reaction steps that often compromise final product purity. Moreover, these methods can be incompatible with amino acids that are labile to the harsh chemicals employed.^53^

Monoacyl 2-alkyl gem-diamines are key for delivering PMRI peptides.^55^ Their incorporation, or that of 2-alkylmalonyl residues, allows inversion of peptide bond directionality without affecting terminal groups.^54^ Optically pure monoacyl 2-alkyl gem-diamines can be obtained from peptide amides using [bis­(trifluoroacetoxy)­iodo]­benzene (TIB), a mild Hofmann-type rearrangement introduced by Loudon et al., and later shown to proceed without racemization by Goodman and Pallai.^55^ However, some derivatives are unstable and may hydrolyze, and the oxidative nature of TIB restricts its use with oxidation-sensitive amino acids, though *t*Bu protecting groups remain stable.^56^ When coupling is inefficient, malonate residues provide an alternative.^54^ Due to gem-diamine instability in solution, solid-phase strategies have been developed; notably, Fehrentz et al. anchored amino acids to a resin via the amine, enabling on-resin Hofmann rearrangement of amide-terminated substrates and subsequent derivatization.^57^

OBIMAP circumvents these limitations by enabling the synthesis of partially modified retro peptides (PMR)—featuring reversed amide bond direction—without requiring reagents beyond those used in conventional SPPS. As a proof of concept, a peptide was successfully synthesized with this reversed backbone architecture. Importantly, this methodology provides a versatile platform by readily incorporating amino acids of alternative stereochemistry, and it can be directly extended to the synthesis of PMRI peptides through the use of inverted chirality to construct the second chain (Figure 5).

**Figure 5:**
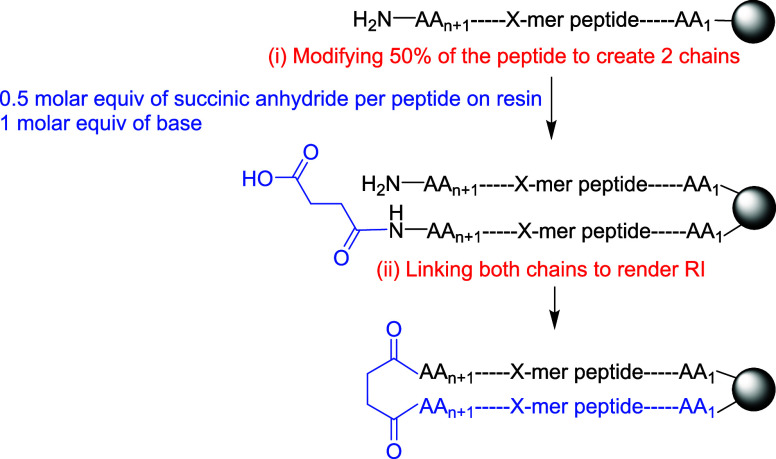
Schematic representation of synthesizing partial retro peptides in two steps using an OBIMAP.

The synthesis of the PMR peptide commenced with the short pentapeptide H-YGFGL-NH_2_ immobilized on a Siber-amide resin (Figure S18). The *N*-termini of the peptide chains were then partially modified by treatment with a half-molar equivalent of succinic anhydride relative to the synthesis scale. This strategy yielded a mixed population of peptides: approximately half of the chains were functionalized with a carboxylic acid at their *N*-terminus, while the remainder retained their native amino group. This created complementary reactive sites—carboxylic acids and free amines—on different chains, enabling the subsequent interchain coupling reaction to proceed (Figure S19). Interestingly, diglycolic anhydride can also be employed to functionalize the *N*-terminus, introducing an additional oxygen atom that may act as a hydrogen-bond acceptor and increase the peptide flexibility, potentially enhancing target engagement. Several PMR peptides incorporating this moiety were synthesized in our laboratory and demonstrated to be viable (data not shown).

A 0.5 molar ratio of succinic anhydride to the bead-bound peptide, with 1 equiv of base, was found to be optimal, as it theoretically generates an equimolar mixture of modified and unmodified peptide precursors. This ensures a 1:1 stoichiometry of the bead-bound peptide chains, which is critical for complete consumption of both species during the subsequent interchain assembly reaction, thereby maximizing the yield of the final product. In practice, a slight deviation from this ideal ratio was observed. HPLC analysis revealed a stoichiometry of H-YGFGL-NH_2_ to Succinic-YGFGL-NH_2_ of 0.9:1.1, corresponding to a 9% molar excess of the succinylated peptide, which appeared as unreacted material in the final product mixture. Despite this minor imbalance, the decapeptide was successfully synthesized in just two steps: initial *N*-terminal modification followed by interchain assembly.

HPLC analysis of the reaction mixture (Figure S20) confirmed the successful formation of the products. MS further verified the identity of the 4-oxobutanoic acid-modified intermediate and the target PMR peptide (Figures S21 and S22).

To further demonstrate the utility of this efficient two-step method, a fragment (residues 27–36) of the HIV fusion inhibitor Enfuvirtide (T-20, Fuzeon)—a 36-amino acid peptide drug approved by the U.S. FDA in 2003—was used as the template to synthesize a corresponding PMR analogue (Figure S23).^58^ While traditional methods for preparing a 20-amino acid peptide are notoriously inefficient—suffering from long synthesis times, low yields, and poor purity—OBIMAP circumvented these limitations. This peptide was synthesized in merely two steps with high purity, equivalent to that of its starting materials (Figure S24). The expected mass was verified by MS (Figure S25).

To further demonstrate the efficiency and scalability of our protocol, it was applied to the synthesis of a 60-amino acid peptide. This target was based on TD2.2, a reported 30-mer blood–brain barrier (BBB) shuttle peptide that specifically targets oligodendrocytes without engaging nonglial cells, such as human neural cells or dermal fibroblasts.^59,60^ The peptide chain was prepared using a microwave-assisted automatic synthesizer.^61^ Following the same procedure established for the T-20 peptide fragment, the *N*-terminus was subsequently modified with succinic anhydride to introduce the necessary functionality for the interchain assembly reaction (Figure S26). Notably, this complex 60-mer was synthesized with high efficiency, achieving a purity on par with the simpler precursor chains (Figure S27). The successful formation of the target molecule was further verified by MS (Figure S28).

The incorporation of the sterically hindered α-aminoisobutyric acid (Aib) residue is of considerable importance. Aib is a nonproteinogenic amino acid frequently employed in pharmaceutical development to confer resistance to degradation by dipeptidyl peptidase-4 (DPP-4)^62^ and has been shown to dramatically enhance the metabolic stability of various GLP-1 analogues, including semaglutide.^63^ However, its incorporation presents a recognized challenge in peptide synthesis and is often used as a benchmark to evaluate the efficiency of new coupling methodologies.^64,65^ This difficulty is also evident in the peptide precursor, where an Aib-containing peptide—synthesized on a microwave-assisted automated synthesizer—shows reduced purity. In a striking demonstration of OBIMAP’s efficiency, a 60-mer PMR peptide was successfully prepared containing eight Aib residues in merely two reaction steps. This represents a significant advancement in SPPS for two key reasons. First, conventional stepwise SPPS typically fails to produce peptides of this length, necessitating complex convergent or hybrid (solid-phase and solution-phase) strategies that introduce additional steps, cost, and yield losses.^60^ Second, OBIMAP surmounts the inherent difficulty of incorporating multiple Aib residues, achieving through a streamlined, two-step process what is notoriously challenging even for automated synthesizers.

### 2.9 Ultrashort Cyclic Peptides

OBIMAP was further leveraged to synthesize exceptionally small cyclic peptides. To the best of our knowledge, the resulting two-amino acid analogue represents the shortest cyclic peptide synthesized to date (Figure S29), a structure impossible to access via conventional SPPS other than diketopiperazine.^66^ Diketopiperazine is a side reaction influenced by several factors (sequence, conformation, among others);^67^ on the other hand, OBIMAP offers the design and synthesis of diverse, unreported cyclic dipeptides, an interesting class that is very stable and able to bind to a wide range of receptors.^66^ For example, cyclic dipeptide synthesis utilizing Asp or Glu with any of Lys/Orn/Dab/Dap, also Cys with Cys, which cannot be cyclized otherwise with standard SPPS or in solution.

The synthesis was achieved as follows: Asp and Lys were first incorporated onto the Pam resin. Their side chains were then linked together to form the initial constraint. Subsequently, 50% of the peptide chains were selectively functionalized at their *N*-termini with succinic anhydride, converting the amine to a carboxylic acid (as detailed in the PMR Section 2.8). Finally, an interchain reaction between the modified (carboxylic acid) and unmodified (amine) *N*-termini yielded the novel cyclic product depicted in Figure S29. The mass of this cyclic peptide was confirmed by MS (Figure S30).

### 2.10 Current Industrial Peptide Families

To demonstrate its industrial applicability, OBIMAP was evaluated for the assembly of a complex therapeutic peptide. This investigation yielded advancements in two key areas: first, the method enables the convergent synthesis of two entirely dissimilar peptide chains. This was achieved by leveraging the orthogonality between the Boc and Fmoc reaction strategies. The Boc-based chain was assembled first, as its side-chain protecting groups are stable to the trifluoroacetic acid (TFA) required for Boc deprotection during elongation. Subsequently, the Fmoc-based chain was synthesized on the same bead. Second, the interchain assembly reaction was designed to connect two distal sites: specifically, an *N*-terminus to a *C*-terminus.

The glucagon-like peptide-1 (GLP-1) analogue dulaglutide (HGEGTFTSDVSSYLEEQAAKEFIAWLVKGGG, 31 amino acids) was selected as a model system to probe this application. The sequence was strategically split into four fragments. These were assembled pairwise on a single bead as Boc- and Fmoc-chemistry chains, setting the stage for the key ligation reaction (Figure S31). The four fragments were strategically designed such that the Fmoc chain contained an amino acid with a reactive side chain. This allowed the chain to be anchored to the resin via its side chain, thereby leaving its *C*-terminus freely available for subsequent ligation with the *N*-terminus of the Boc chain. For the sake of clarity, the following discussion will focus on the assembly of the first two chains as a representative example.

Following the independent assembly of both chains, an interchain ligation reaction was performed. The desired product was successfully obtained as the major species with satisfactory purity, as shown in Figure S32. The identities of all peptide products were confirmed by MS (Figure S33).

These findings demonstrate that the OBIMAP successfully facilitates the linkage of distal residues through the interchain assembly reaction. Consequently, the OBIMAP method provides an efficient route to synthesize complex industrial peptides, such as dulaglutide, with high selectivity—effectively minimizing side products and achieving a final product of satisfactory purity.

### 2.11 Cross-Linked Peptide

To validate the feasibility of assembling three distinct peptide chains on a single solid support, we leveraged the OBIMAP platform to synthesize a complex, triply cross-linked peptide. The assembly was meticulously designed to maintain orthogonality throughout the synthesis. The chemical structure of this novel multibranched peptide is presented in Figure 6.

**Figure 6:**
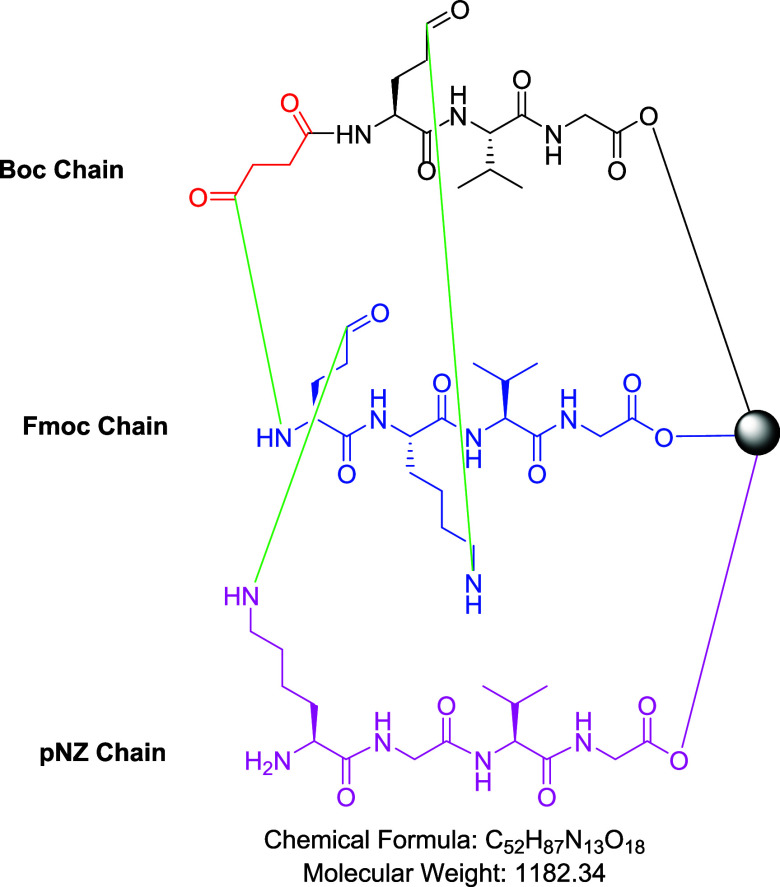
Chemical structure of a novel triple cross-linked peptide. Green: the formed peptide bond.

The first and second chains were interconnected through a dual-linkage strategy: one bond was formed between the active side chains of Lys and Glu residues, while a second distinct bond was created between their *N*-termini following the selective modification of one terminus with succinic anhydride, as previously detailed in Section 2.8. A third interchain bond was subsequently formed between the second and third chains via the side chains of an additional Lys and Glu residue pair. It is worth noting that the assembly of the three chains was carefully designed. The first two chains were constructed using one-third of the available reactive sites each, employing Boc and Fmoc strategies, while reserving the remaining third of the sites on the resin for the third chain. These reserved sites were protected with pNZ, which is stable under both acidic and basic conditions. Following assembly and on-demand cross-linking of the first two chains, the pNZ group was removed to expose the reserved reactive sites, allowing elongation of the third chain and subsequent cross-linking at the required positions. The final peptide was then cleaved from the resin and fully characterized. The target peptide was obtained in satisfactory purity (Figure S34), and its molecular mass was confirmed by MS (Figure S35).

These innovative structures are anticipated to exhibit exceptional conformational rigidity, metabolic stability, and target engagement. By effectively circumventing the primary obstacle of enzymatic degradation, OBIMAP significantly enhances the potential for developing viable peptide therapeutics. To the best of our knowledge, this represents a first-in-class strategy for peptide design with no prior precedent.

The architecture draws inspiration from double-stranded oligonucleotides, which employ complementary sense and antisense strands.^68^ Similarly, our cross-linked peptides can be engineered so that each individual chain possesses a distinct and independent biological function. This design enables the creation of synergistic and multifunctional therapeutics. A particularly powerful advantage of this approach is the capacity to selectively modify one chain to optimize its activity or pharmacokinetic profile without disrupting the function of its partnered chain(s). A key advantage of this architecture is that each constituent chain can be independently optimized for critical pharmacokinetic properties—such as log *P*, log *D*, and p*K*_a_—which are paramount for achieving oral bioavailability yet remain notoriously difficult to engineer in conventional peptides. Furthermore, the constrained, multichain design confers unprecedented resistance to enzymatic degradation. This combination of independently tunable properties and innate stability makes this novel class of peptides a highly promising platform for the development of orally delivered therapeutics.

Given the disulfide-linked two-chain architecture of insulin, the ability of OBIMAP to generate cross-linked peptide assemblies suggests its potential to enable fully solid-phase synthesis of insulin.

## 3 Conclusion

The technology presented in this work, OBIMAP, establishes a new paradigm for peptide synthesis, significantly expanding the peptide reaction space beyond traditional SPPS. By enabling the concurrent assembly of different peptide chains on a solid support and their subsequent linkage via a solid-phase interchain reaction, OBIMAP provides access to novel and significantly new peptide families that are difficult or impossible to obtain using conventional methods. This capability is exemplified by our successful synthesis of highly constrained ultrashort cyclic tetrapeptides and dipeptides, architectures previously unattainable through standard SPPS. Furthermore, it has been demonstrated that OBIMAP enables the efficient, two-step synthesis of PMR peptides without the need for aggressive chemicals that can compromise amino acid integrity. The robustness of OBIMAP is further highlighted by its ability to deliver a challenging 60-mer peptide with high efficiency and purity, overcoming the well-known limitations of synthesizing long peptides. Its applicability to industrial production is confirmed by the successful preparation of fragments for therapeutic peptides, such as dulaglutide, with satisfactory purity.

Hybrid manufacturing approaches for peptide therapeutics have required extensive development efforts to address fragment design, solubility, and purity.^25^ By contrast, OBIMAP is rooted in more than 60 years of established academic and industrial experience with SPPS. Scale-up across technology readiness levels (TRLs) 3–9 is well documented for conventional SPPS and is therefore expected to be directly transferable to this SPPS-based platform. As the core principles of SPPS are retained, scale-up to gram-scale production is anticipated to be readily achievable. In a manuscript currently under review, our research group has successfully applied the OBIMAP technology to the fully on-resin synthesis of liraglutide, including the lipidation step, with comprehensive optimization and evaluation of both purity and yield. This work demonstrates the superior performance of OBIMAP compared to conventional Merrifield methodology, achieving higher product purity and overall yield.

Looking forward, the potential of OBIMAP to address a critical challenge in peptide therapeutics—oral bioavailability—is particularly compelling. Of the 22 US FDA-approved peptides in the past decade, only 4 are administered orally.^69^ Given the recognized enhanced metabolic stability of cyclic and cross-linked structures, we plan to leverage OBIMAP to generate novel cross-linked peptide analogues. It is anticipated that these new designs and structural motifs will exhibit unprecedented stability and extended half-lives, thereby offering a promising strategy to overcome the oral delivery limitations that hinder many current peptide-based drugs.

## 4 Materials and Methods

PuroSynth CTC (1.0 mmol/g, supplier’s specification) and PuroSynth Rink Amide (0.5 mmol/g, supplier’s specification) resins were used for all syntheses. All reagents and solvents were obtained from commercial suppliers and were used without further purification unless otherwise stated. Analytical HPLC was performed on a Shimadzu LC20 system using Lab Solution software for data processing. Column: Symmetry Luna C18 (3.6 μm, 4.6 × 150 mm) column, with a flow rate of 1.0 mL/min and UV detection at 220 nm. Mobile phase A was 0.1% trifluoroacetic acid (TFA) in H_2_O, and mobile phase B was 0.1% TFA in CH_3_CN. Mass analysis was done using a Velos Pro mass spectrometer (ThermoFisher Scientific), a hybrid linear trap quadrupole (LTQ)-Orbitrap, using positive electrospray ionization mass spectrometry (ESI ± MS) by direct infusion of samples. Automatic peptide synthesis was done using Liberty Blue automated microwave-assisted peptide synthesizer (CEM).

### 4.1 Incorporation Procedure

#### 4.1.1 CTC Resin

First, amino acids were incorporated onto CTC resin using dry CH_2_Cl_2_. The CTC resin was swelled in CH_2_Cl_2_ for 10–20 min. The Fmoc-amino acids (2 equiv) were dissolved in a minimum amount of CH_2_Cl_2_ (0.5 mL/100 mg resin) and sonicated for 10 min. *N,N*-Diisopropylethylamine (DIEA) (4 equiv) was then added to the solution, which in turn was added to the previously swelled resin and allowed to react for 1 h under mechanical shaking. After this, MeOH (80 μL/100 mg of resin) was added to end-cap any unreacted chloride of the CTC resin. Finally, the resin was washed twice with CH_2_Cl_2_ and dried under vacuum.

#### 4.1.2 Rink Amide and Sieber Amide Resins

First, amino acids were incorporated onto Rink Amide resin using dry DMF. The Rink Amide resin was swelled in DMF for 10–20 min. Fmoc was removed using 20% piperidine/DMF, and the mixture was allowed to shake for 2 and 7 min with a fresh quantity of the 20% piperidine/DMF. The Fmoc-amino acids (3 equiv) and OxymaPure (3 equiv) were dissolved in a minimum amount of DMF (0.5 mL/100 mg resin) and sonicated for 10 min. *N,N*-diisopropylcarbodiimide (DIC) (3 equiv) was then added to the solution, which, in turn, was added to the previously swelled resin and allowed to react for 1 h under mechanical shaking. Finally, the resin was washed twice with CH_2_Cl_2_ and dried under vacuum.

#### 4.1.3 Pam Resin

First, amino acids were incorporated onto Pam resin using dry CH_2_Cl_2_. CTC resin was swelled in CH_2_Cl_2_ for 10–20 min. 4-Dimethylaminopyridine (DMAP) (0.25 equiv) was then added to the resin. The Fmoc-amino acids (2.5 equiv) were dissolved in a minimum amount of CH_2_Cl_2_ (0.5 mL/100 mg resin) and sonicated for 10 min. DIC (5 equiv) was then added to the solution, which in turn was added to the previously swelled resin and allowed to react for 1 h under mechanical shaking. After this, acetic anhydride and DIEA (10:20) in DMF were added to end-cap any unreacted OH groups of the Pam resin. Finally, the resin was washed twice with CH_2_Cl_2_ and dried under vacuum.

#### 4.1.4 Reactive Amino Acids Incorporation (Asp and Lys)

When coupling Asp and Lys to the resin as key amino acids for the subsequent interchain assembly reaction, 33% Asp and 67% Lys are considered the optimum ratio that ensures complete conversion. The incorporation of these amino acids was performed twice to ensure their full incorporation into the peptide chain.

### 4.2 Peptide Synthesis

Peptides were synthesized following the standard methodology performed in our laboratory, using 3 equiv of Fmoc-AA–OH, 3 equiv of OxymaPure, and 3 equiv of DIC or 2 equiv of Fmoc-AA–OH, 1.9 equiv of (1-cyano-2-ethoxy-2-oxoethylidenaminooxy)­dimethylamino-morpholino-carbenium hexafluorophosphate (COMU), and 4 equiv of DIEA in DMF, and then the mixture was shaken for 1 h. Fmoc was then removed (see t Section 4.1.2). All Arg and the residue that comes after were double-coupled to ensure complete coupling.

### 4.3 Allyl Protecting Group Cleavage

To deprotect Asp­(OAll) or Lys­(Alloc), tetrakis­(triphenylphosphine)­palladium(0) (0.1 equiv) and triphenylsilane (10 equiv) in CH_2_Cl_2_ were added to the peptidyl resin and allowed to react for 1 h under mechanical shaking. This step was done twice. The resin was washed twice with CH_2_Cl_2_ and then with *N*,*N*-diethyltithiocarbamate (0.02 M in DMF) three times to wash the Pd out from the resin.

### 4.4 Boc, Trt, *t*Bu Protecting Group Cleavage

To cleave Boc, Trt, or *t*Bu protecting groups, 50%TFA in CH_2_Cl_2_ was added to the peptidyl resin and allowed to react for 30 min under mechanical shaking. The resin was washed twice with CH_2_Cl_2_ and then treated with 10% DIEA in CH_2_Cl_2_ and allowed to react for 30 min under mechanical shaking to neutralize the protonated groups due to acid treatment. Finally, the resin was washed twice with CH_2_Cl_2_ and dried under vacuum.

### 4.5 Interchain Assembly Reaction

Once the reactive amino acids are ready to form the amide bond, 3 equiv of Fmoc-AA–OH, 3 equiv of OxymaPure, 3 equiv of DIC or 2 equiv of Fmoc-AA–OH, 1.9 equiv of (1-cyano-2-ethoxy-2-oxoethylidenaminooxy)­dimethylamino-morpholino-carbenium hexafluorophosphate (COMU), 4 equiv of DIEA in DMF are used, followed by shaking for 24 h.

### 4.6 Automatic Synthesis

Within the automated SPPS method, coupling of the amino acid to the growing peptide chain was achieved through the addition and heating of the Fmoc-AA–OH acid (0.25 mmol, 5 equiv, 0.2 M in DMF), OxymaPure (0.25 mmol, 5 equiv, 0.5 M in DMF), and DIC (0.50 mmol, 10 equiv, 0.5 M in DMF) at 90 °̊C for 2 min (single coupling) or 2 × 2 min (double coupling). *N*-terminal deprotection of the growing peptide chains was achieved through Fmoc-cleavage via the addition of piperidine (20% v/v in DMF) and OxymaPure (0.1 M in DMF) and heating at 90 °C for 1.5 min.

### 4.7 Cleavage Protocols

#### 4.7.1 Rink Amide and CTC Resins

The final synthesized peptide was cleaved from the resin using TFA/triisopropylsilane (TIS)/H_2_O (95:2.5:2.5) (1 mL/100 mg) under mechanical shaking for 1 h. Chilled diethyl ether was then added (5 times the cleavage solution volume), and the solution was kept in an ice bath for 30 min. The solution was then centrifuged for 5 min at 5000 rpm, and the supernatant was decanted. A new amount of the ether (5 times the cleavage solution volume) was added to repeat this step. Any remaining ether was dried under N_2_. Finally, the precipitate was dissolved in CH_3_CN–H_2_O (1:1). A small amount of the solution was injected into the HPLC system to check the purity of the final product.

#### 4.7.2 Pam Resin

The final synthesized peptide was cleaved from the resin using triflic acid/TFA/TIS (8:3:1) (1 mL/100 mg) under mechanical shaking for 1 h at 0 °C. Chilled diethyl ether was then added (5 times the cleavage solution volume), and the solution was kept in an ice bath for 30 min. The solution was then centrifuged for 5 min at 5000 rpm, and the supernatant was decanted. A new amount of the ether (5 times the cleavage solution volume) was added to repeat this step. Any remaining ether was dried under N_2_. Finally, the precipitate was dissolved in CH_3_CN–H_2_O (1:1). A small amount of the solution was injected into the HPLC system to check the purity of the final product.

## Supplementary Material



## Notes

Chemical structures, HPLC chromatograms, mass spectrometry spectra (PDF)
