# Supplementary material for: OBIMAP (One-Bead Interchain Multipeptide Assembly Platform)
Source: ACS Bio Med Chem Au. 2026 Jan 14;6(1):90–100. doi: 10.1021/acsbiomedchemau.5c00237 (PMC12921515; doi:10.1021/acsbiomedchemau.5c00237)
Supplement: Supplementary file 1 [file bg5c00237_si_001.pdf]

OBIMAP (One-Bead Interchain Multi-Peptide Assembly Platform)

Othman Al Musaimi[1,2,3.4*], and Daryl R. Williams[3,4]

1. School of Pharmacy, Newcastle University, Newcastle upon Tyne, NE1 7RU, UK

2. Translational and Clinical Research Institute, Faculty of Medical Sciences, Newcastle University, Newcastle upon Tyne NE1 7RU, UK

3. Department of Chemical Engineering, Imperial College London, London, SW7 2AZ, UK

4. Orthogonal Peptides Limited, London, SW7 2AZ, UK

Correspondence: OAM, othman.almusaimi@newcastle.ac.uk

Supplementary Figure 1. Schematic diagram of linear peptide synthesis following interchain assembly reaction Fmoc-GDGL-OH and Fmoc-GKGL-OH.

Supplementary Figure 2. Chromatograms of linear octapeptide formed from Fmoc-GDGL-OH and Fmoc-GKGL-OH.

Supplementary Figure 3. Mass of linear octapeptide formed from Fmoc-GDGL-OH and Fmoc-GKGL-OH.

Supplementary Figure 4. Mass of unreacted Fmoc-GDGL-OH tetrapeptide.

Supplementary Figure 5. Chromatograms of: black: linear Fmoc-Lys-Gly-OH + Fmoc-Asp-Gly-OH incorporated on CTC resin following 50% loading of Asp and 50% of Lys.

Supplementary Figure 6. Chromatograms of: black: linear Fmoc-Lys-Gly-OH + Fmoc-Asp-Gly-OH incorporated on CTC resin following 33% loading of Asp and 67% of Lys.

Supplementary Figure 7. Schematic representation of cyclic peptide synthesis following interchain assembly reaction.

Supplementary Figure 8. Chromatograms of cyclic Fmoc-DK-G-G-DK-OH peptide (t_R= 18,2 min).

Supplementary Figure 9. Mass of cyclic peptide.

Supplementary Figure 10. Chemical structure of a cyclic peptide on rink amide resin.

Supplementary Figure 11. Chromatograms of cyclic Fmoc-DK-G-G-DK-NH_2 peptide.

Supplementary Figure 12. Mass of cyclic peptide.

Supplementary Figure 13. Chromatograms of bicyclic H-DK-L-A-F-DK-G-G-DK-NH_2 peptide.

Supplementary Figure 14. Mass of bicyclic peptide.

Supplementary Figure 15. Chemical structure of a cyclic tetrapeptide on rink amide resin.

Supplementary Figure 16. Chromatograms of cyclic Fmoc-DK-DK-NH_2 peptide.

Supplementary Figure 17. Mass of cyclic tetrapeptide.

Supplementary Figure 18. Mass of YGFGL-NH_2 pentapeptide.

Supplementary Figure 19. Schematic representation of PMR peptide synthesis following interchain assembly reaction.

Supplementary Figure 20. HPLC Chromatograms: black: H-YGFGL-NH_2.

Supplementary Figure 21. Mass of 4-oxobutanoic-YGFGL-NH_2 modified pentapeptide.

Supplementary Figure 22. Mass of H-YGFGL-NH-Succinic-YGFGL-NH_2 decapeptide.

Supplementary Figure 23. Chemical structure of two-fragment (27-36) of Enfuvirtide (T-20 or Fuzeon) linked via succinamide, on Sieber amide resin.

Supplementary Figure 24. Chromatograms of: black: one-fragment (27-36) of Enfuvirtide (T-20 or Fuzeon).

Supplementary Figure 25. Mass of PMR of T-20 fragment.

Supplementary Figure 26. Chemical structure of Aib modified TD2.2 peptide, two-fragment linked via succinamide, on protide resin.

Supplementary Figure 27. Chromatograms of two-fragment of TD2.2-succinicamide-TD2.2.

Supplementary Figure 28. Mass of PMR of modified TD2.2 fragment.

Supplementary Figure 29. Chemical structure of a new 2 amino acid cyclic peptide.

Supplementary Figure 30. Mass of cyclic dipeptide.

Supplementary Figure 31. Chemical structure of the designed dulaglutide fragments.

Supplementary Figure 32. Chromatograms of H-EQAAKEFIAWLVKGGG-OH.

Supplementary Figure 33. Mass of H-EQAAKEFIAWLVKGGG-OH peptide.

Supplementary Figure 34. Chromatograms of the triple cross-linked peptide.

Supplementary Figure 35. Mass of crosslinked peptide.

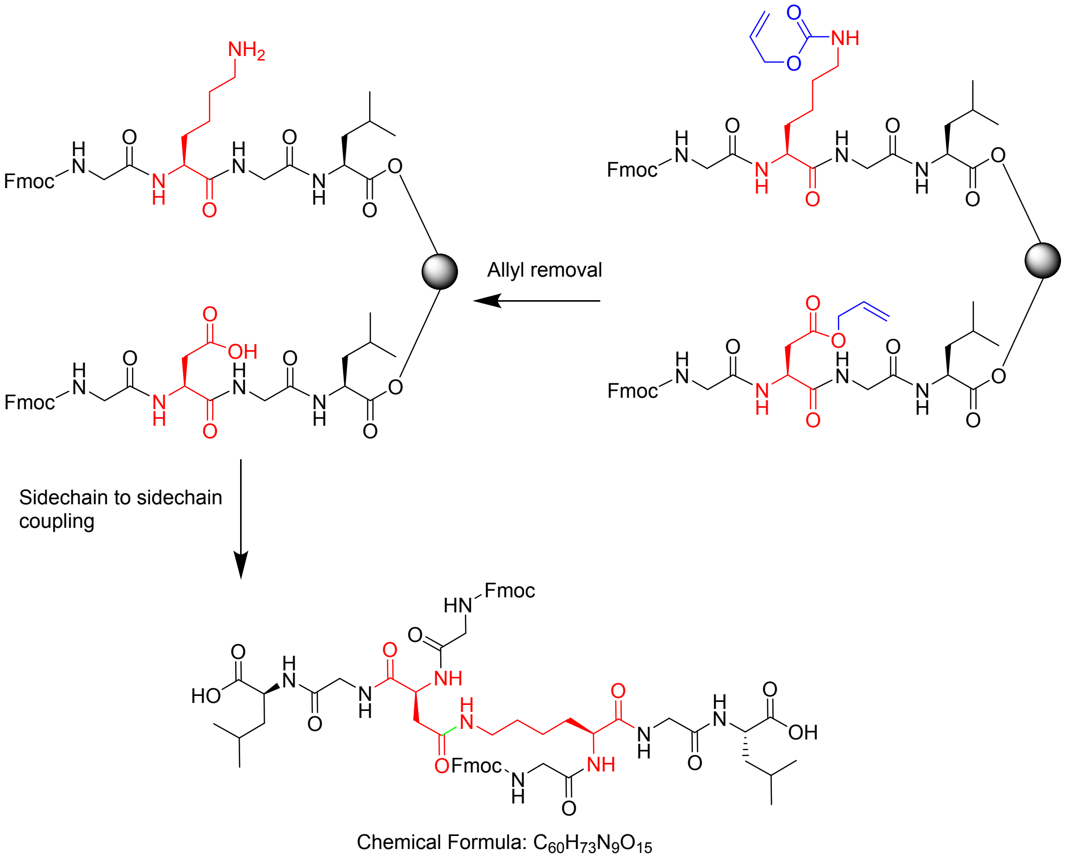

Supplementary Figure 1. Schematic diagram of linear peptide synthesis following interchain assembly reaction Fmoc-GDGL-OH and Fmoc-GKGL-OH. Linking from one joint. Red: Asp and Lys amino acids; blue: allyl/ alloc protecting groups; green: the newly formed isopeptide bond between sidechains.

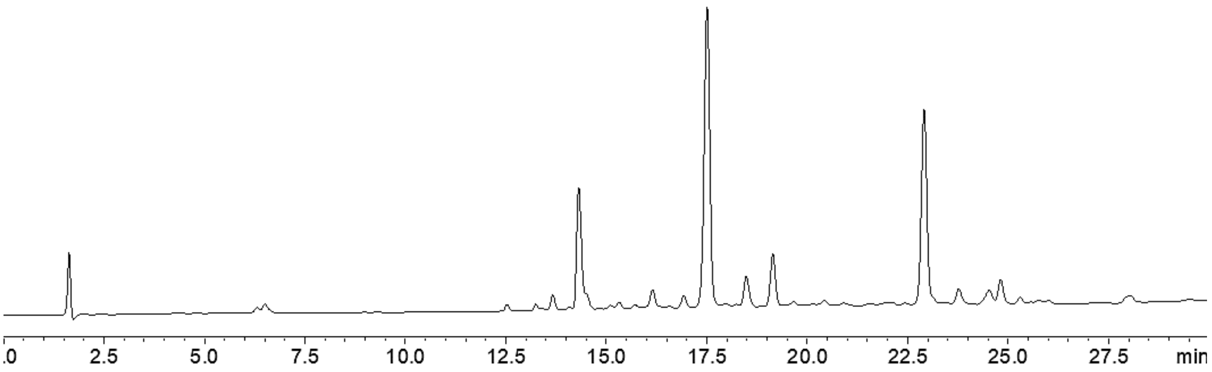

Supplementary Figure 2. Chromatograms of linear octapeptide formed from Fmoc-GDGL-OH and Fmoc-GKGL-OH (t_R= 22,9 min); unreacted Fmoc-GDGL-OH (t_R= 17,5 min). 15–70% in 30 min gradient elution. Mobile phase A: 0.1% TFA in H_2O; mobile phase B: 0.1% TFA in CH_3CN; Symmetry Luna C_{18} (3.6 µm, 4.6 × 150 mm) column.

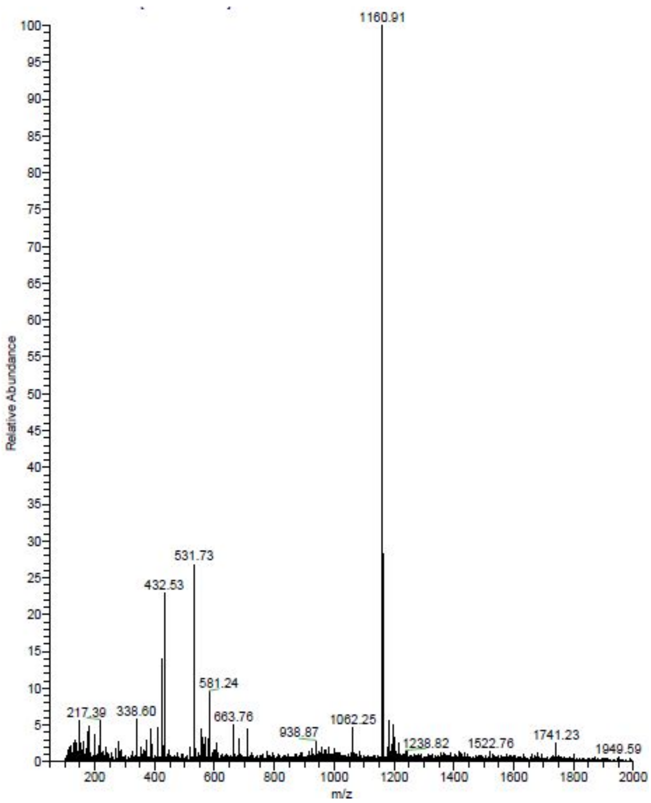

Supplementary Figure 3. Mass of linear octapeptide formed from Fmoc-GDGL-OH and Fmoc-GKGL-OH. Calculated: 1159.52; found: 1160.91 [M+ H]⁺

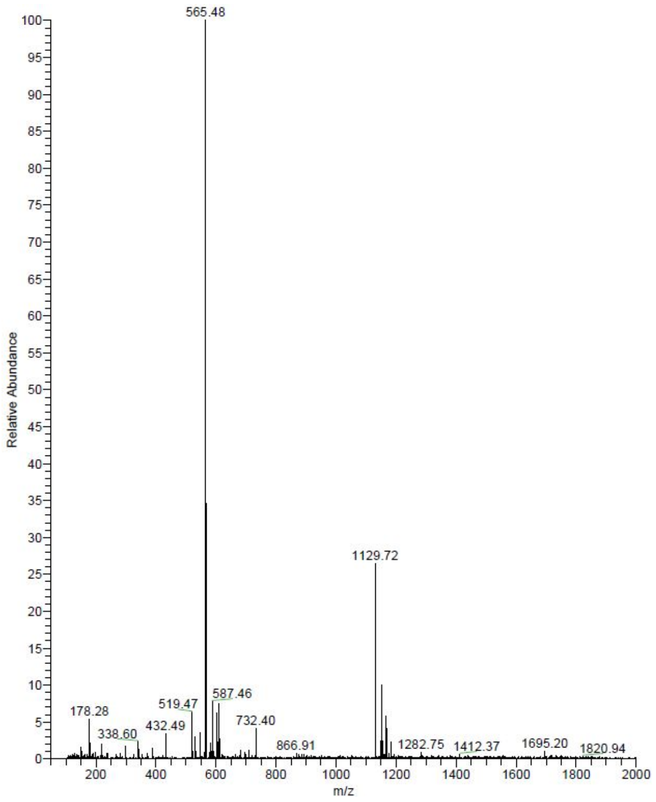

Supplementary Figure 4. Mass of unreacted Fmoc-GDGL-OH tetrapeptide. Calculated: 582.61; found: 565.48 [M-18]⁺

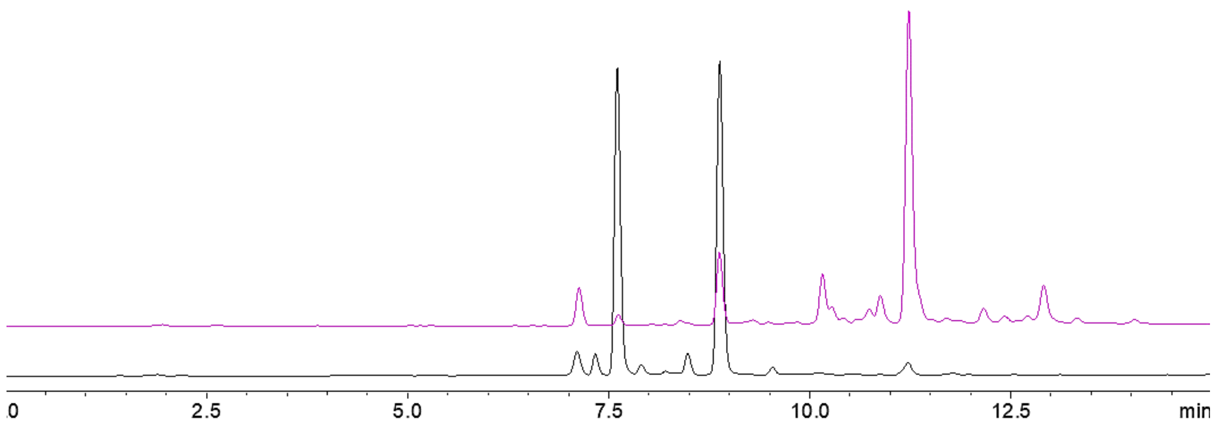

Supplementary Figure 5. Chromatograms of: black: linear Fmoc-Lys-Gly-OH + Fmoc-Asp-Gly-OH incorporated on CTC resin following 50% loading of Asp and 50% of Lys; pink: the product after the reaction between the Asp and Lys for 22h at RT (an excess of the 2nd peak which is Asp is observed). 5−95% in 15 min gradient elution. Refer for the legend of Fig. S2 for the chromatographic conditions.

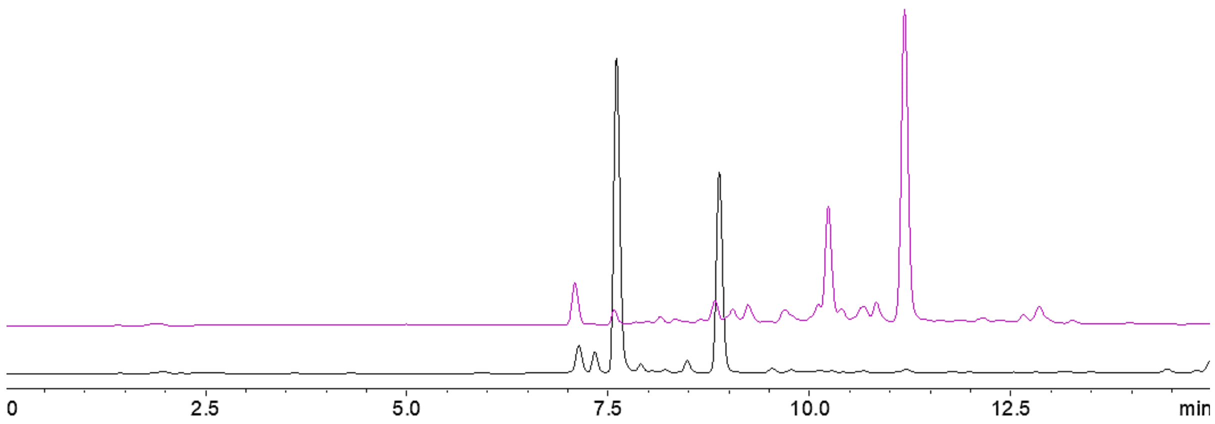

Supplementary Figure 6. Chromatograms of: black: linear Fmoc-Lys-Gly-OH + Fmoc-Asp-Gly-OH incorporated on CTC resin following 33% loading of Asp and 67% of Lys; pink: the product after the reaction between the Asp and Lys for 22h at RT (only 5% of the 2nd peak which is Asp has remained). 5−95% in 15 min gradient elution. Refer for the legend of Fig. S2 for the chromatographic conditions.

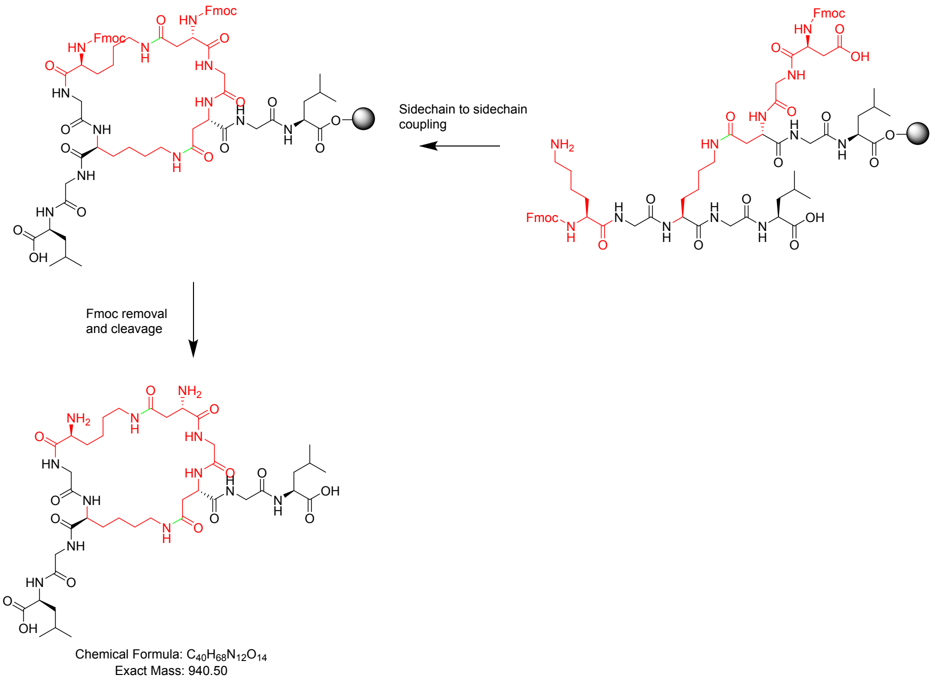

Supplementary Figure 7. Schematic representation of cyclic peptide synthesis following interchain assembly reaction. Linking from two joints. Red: Asp and Lys amino acids; blue: allyl protecting group; green: the newly formed peptide bond.

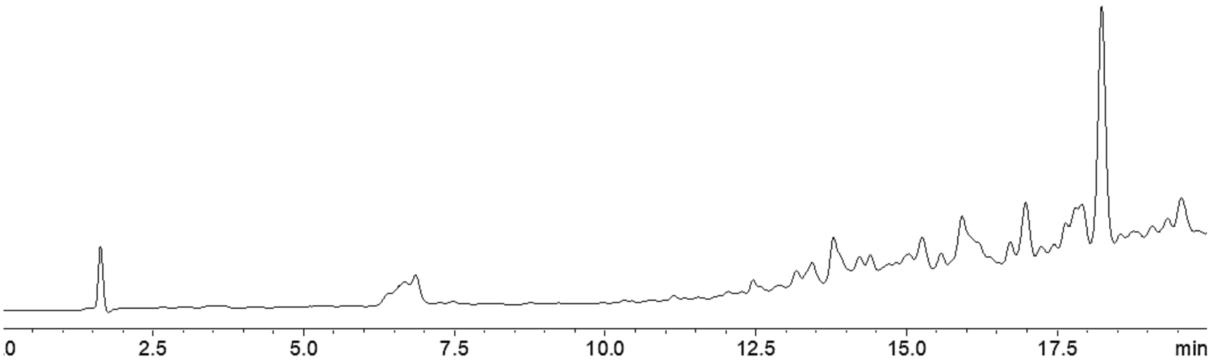

Supplementary Figure 8. Chromatograms of cyclic Fmoc-DK-G-G-DK-OH peptide (t_R= 18,2 min). Refer for the legend of Fig. S2 for the chromatographic conditions.

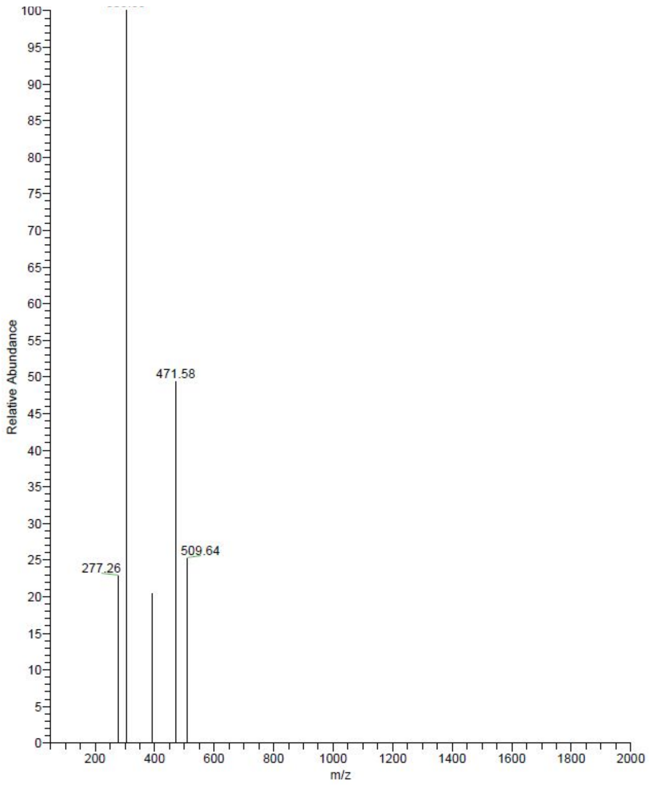

Supplementary Figure 9. Mass of cyclic peptide. Calculated: 940.50; found: 471.58 $[M+2H]^{2+}$

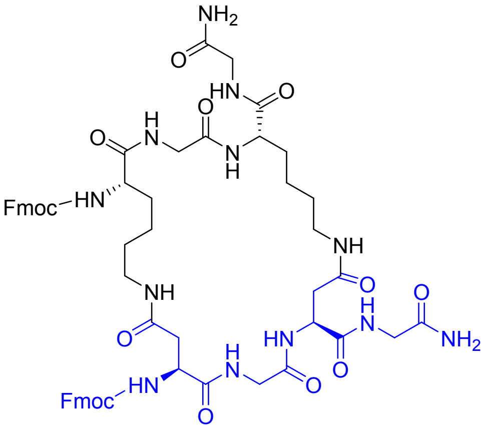

Supplementary Figure 10. Chemical structure of a cyclic peptide on rink amide resin. Black: first chain; blue: second chain.

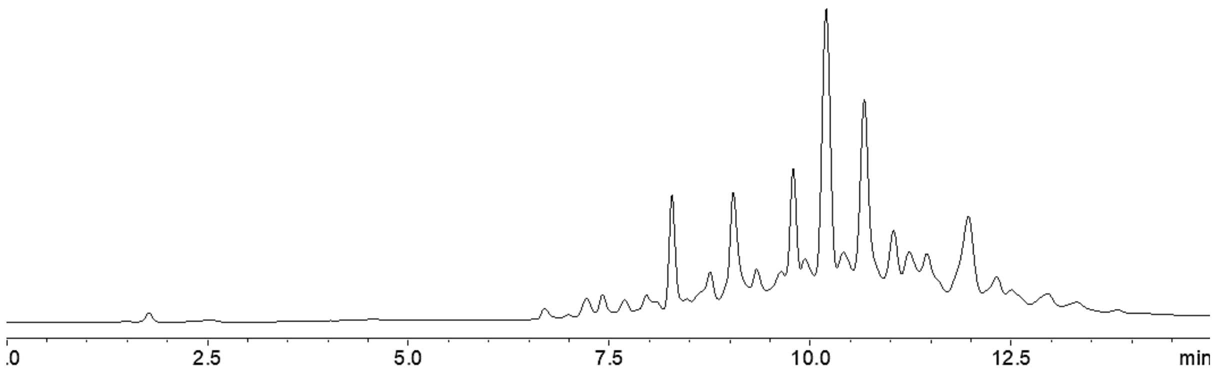

Supplementary Figure 11. Chromatograms of cyclic Fmoc-DK-G-G-DK-NH_2 peptide (t_R= 10,2 min). 5−95% in 15 min gradient elution. Refer for the legend of Fig. S2 for the chromatographic conditions.

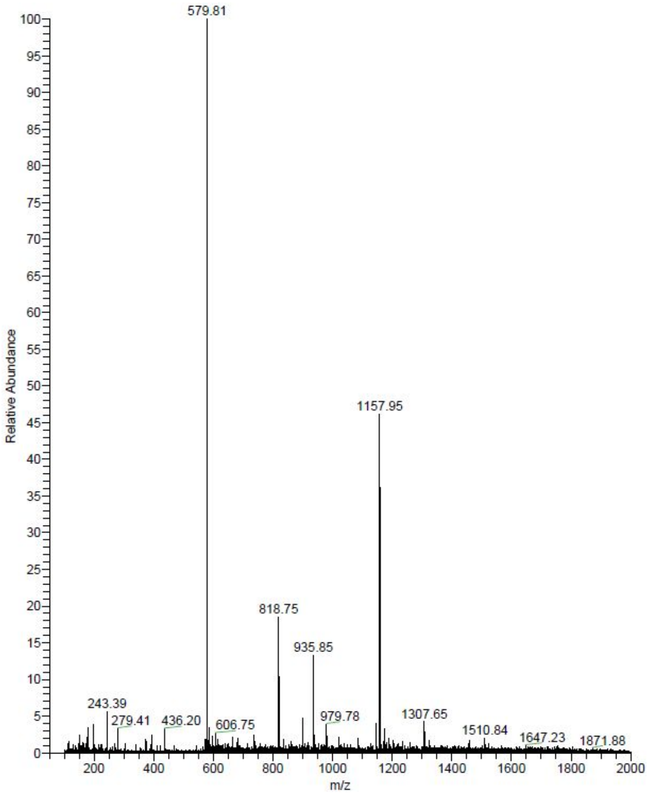

Supplementary Figure 12. Mass of cyclic peptide. Calculated: 1156.50; found: 1157.95 $[M+H]^{+}$, 579.81 $[M+2H]^{2+}$

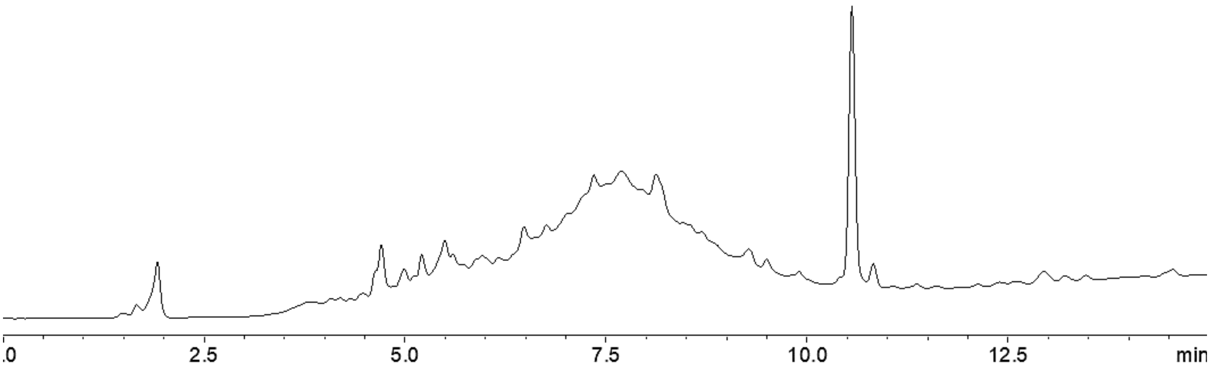

Supplementary Figure 13. Chromatograms of bicyclic H-DK-L-A-F-DK-G-G-DK-NH_2 peptide (t_R= 10,6 min). 5–95% in 15 min gradient elution. Refer for the legend of Fig. S2 for the chromatographic conditions.

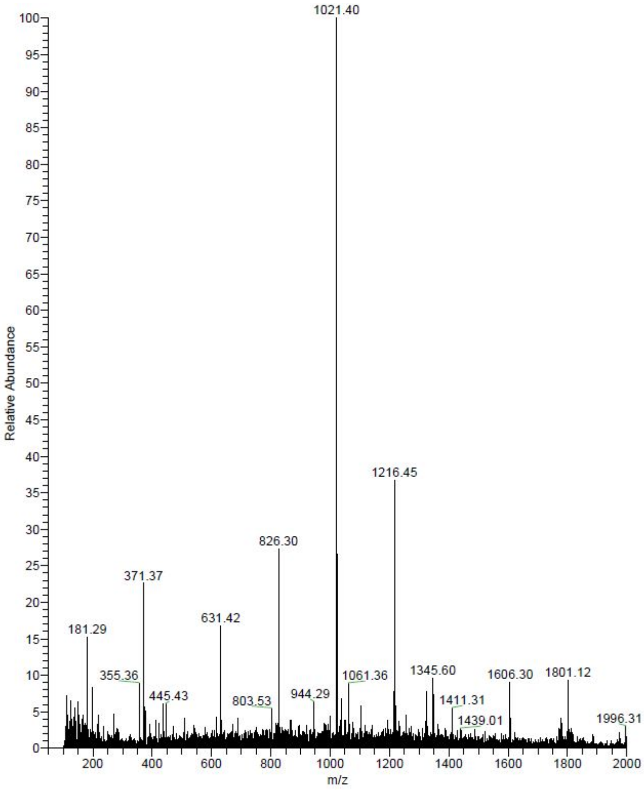

Supplementary Figure 14. Mass of bicyclic peptide. Calculated: 2044.97; found: 1021.40 $[M+2H]^{2+}$

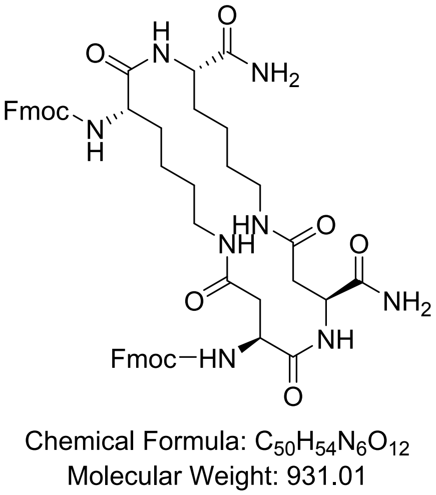

Supplementary Figure 15. Chemical structure of a cyclic tetrapeptide on rink amide resin.

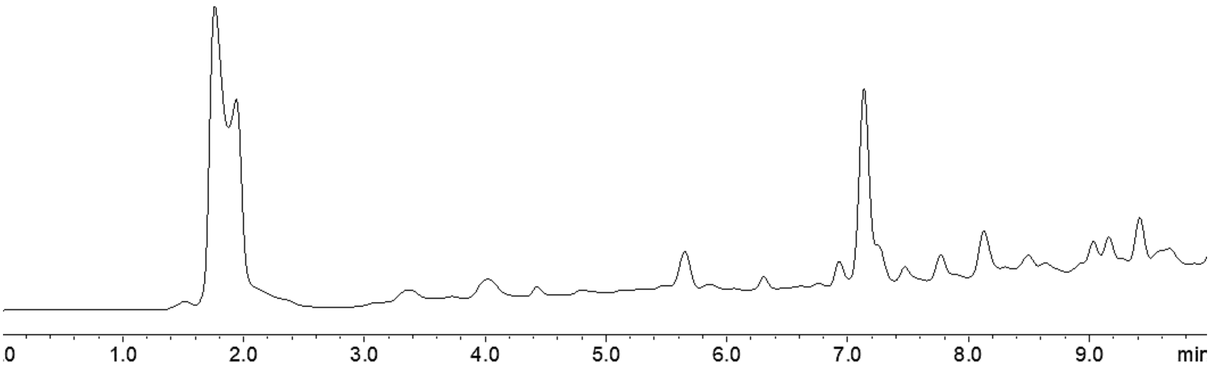

Supplementary Figure 16. Chromatograms of cyclic Fmoc-DK-DK-NH_2 peptide (t_R= 7,1 min). 5–95% in 15 min gradient elution. Refer for the legend of Fig. S2 for the chromatographic conditions.

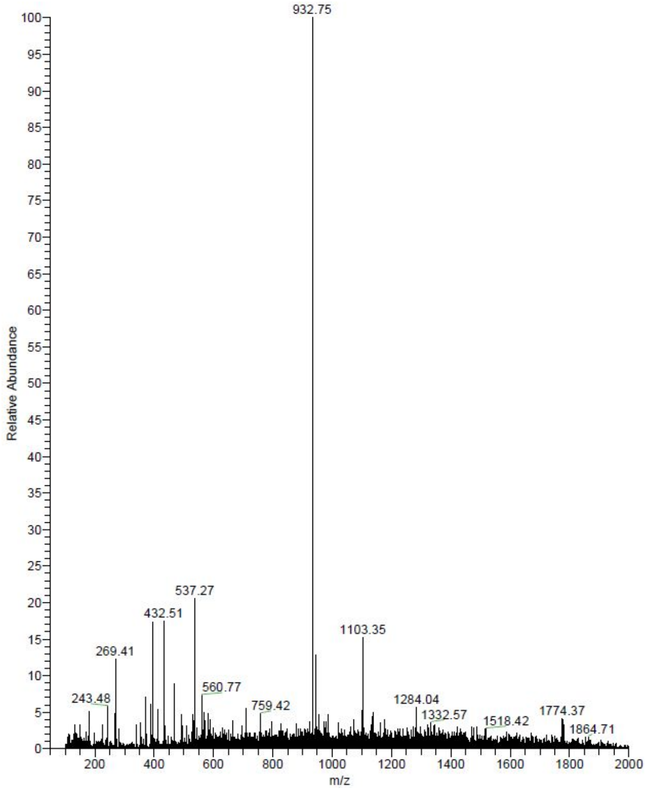

Supplementary Figure 17. Mass of cyclic tetrapeptide. Calculated: 931.01; found: 932.75 [M+ H]$^+$

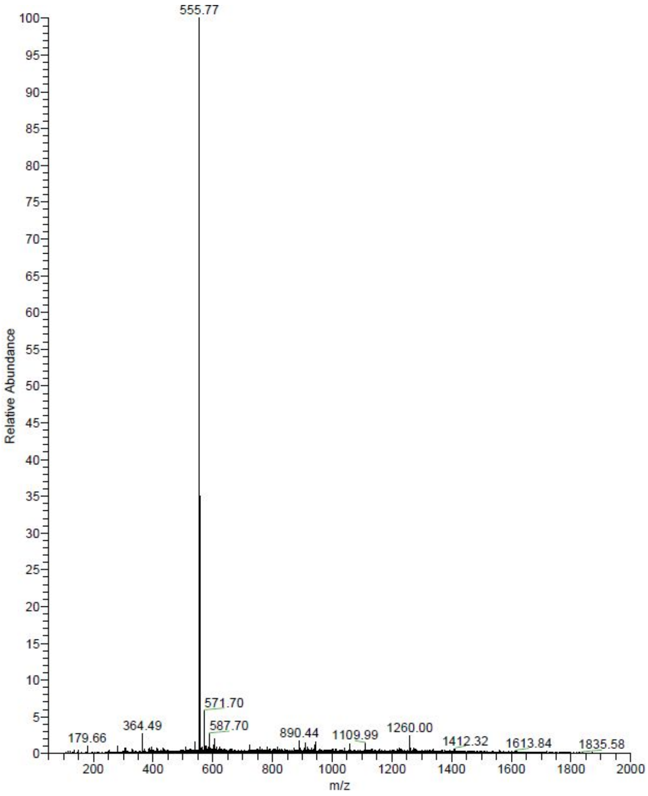

Supplementary Figure 18. Mass of YGFGL-NH_2 pentapeptide. Calculated: 554.29; found: 555.77 [M+ H]$^+$

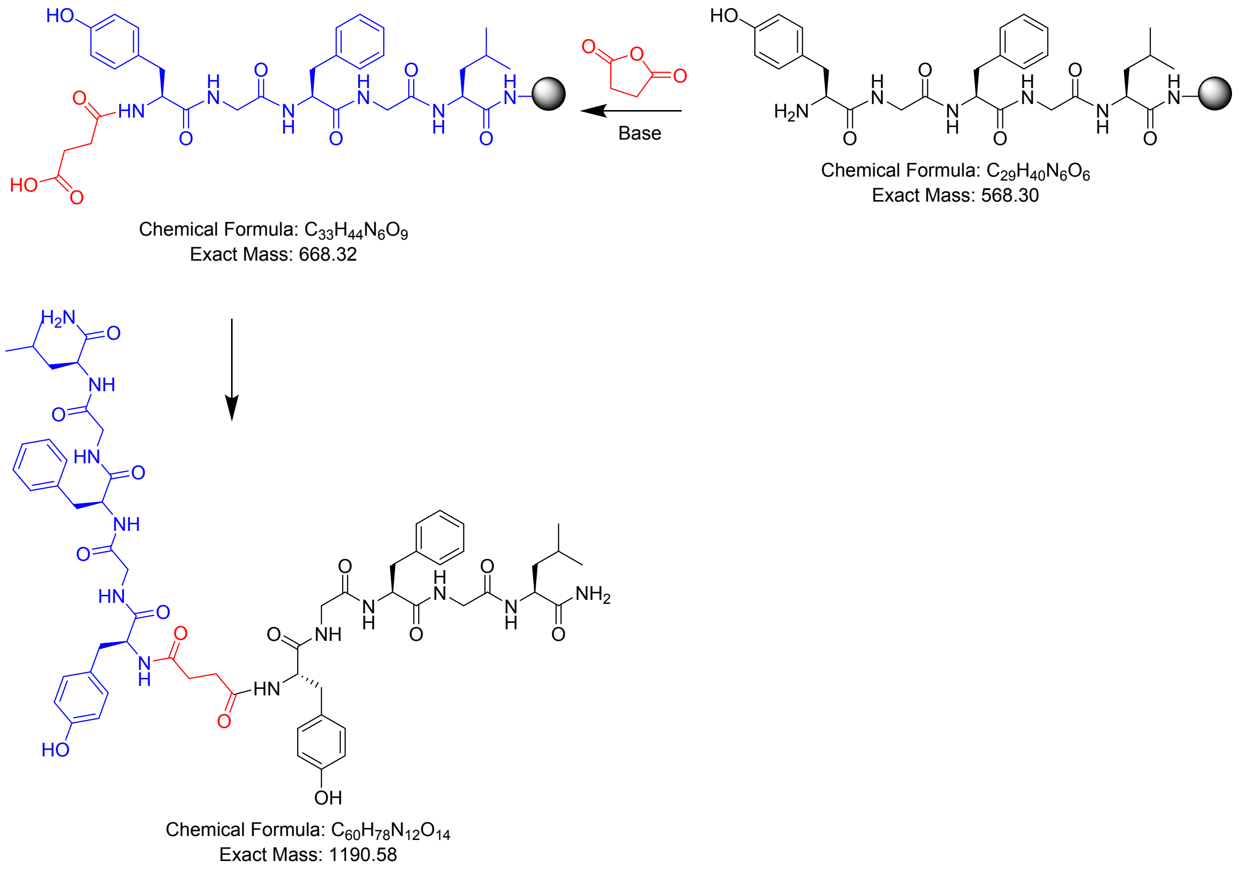

Supplementary Figure 19. Schematic representation of PMR peptide synthesis following interchain assembly reaction. Black: first chain; red: succinic anhydride used to link the two chains; blue: second chain (PMR).

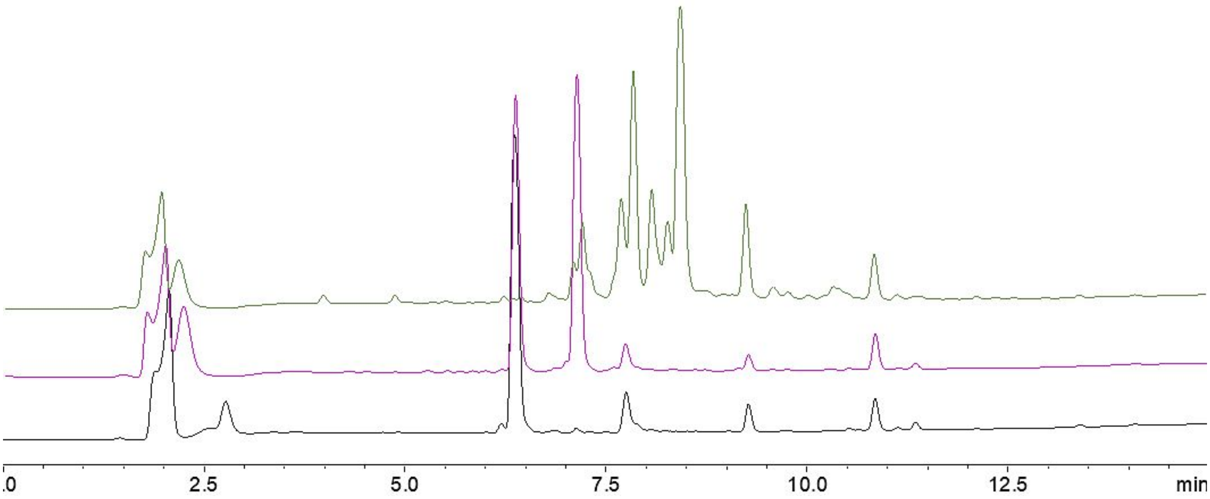

Supplementary Figure 20. HPLC Chromatograms: black: H-YGFGL-NH_2 (t_R= 6,4 min); pink: Succinc-YGFGL-NH_2 (t_R= 7,1 min); green: H-YGFGL-Succ-YGFGL-NH_2 product (t_R= 8,4 min). 5–95% in 15 min gradient elution. Refer for the legend of Fig. S2 for the chromatographic conditions.

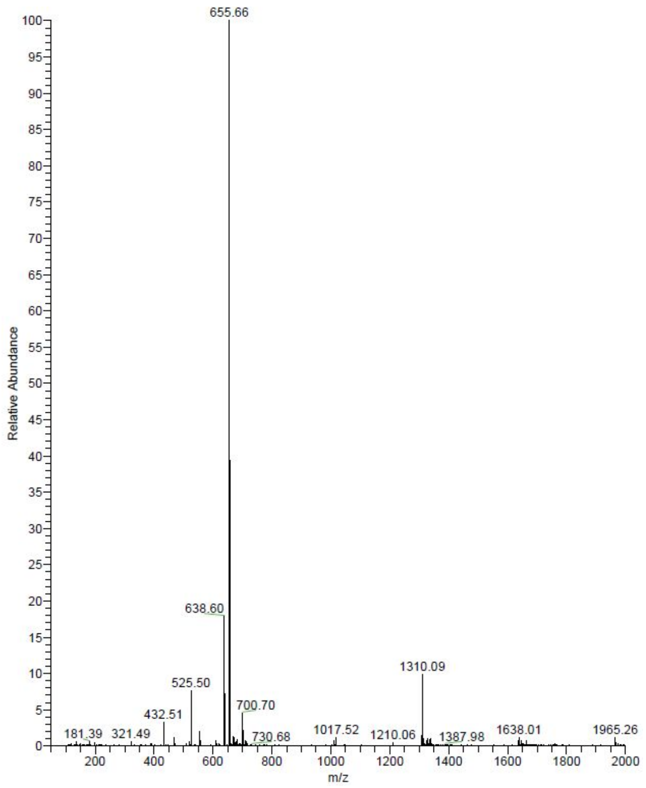

Supplementary Figure 21. Mass of 4-oxobutanoic-YGFGL-NH_2 modified pentapeptide. Calculated: 654.30; found: 655.66 $[M+H]^+$, 1310.09 $[M+2H]^{2+}$ (non-covalent dimer).

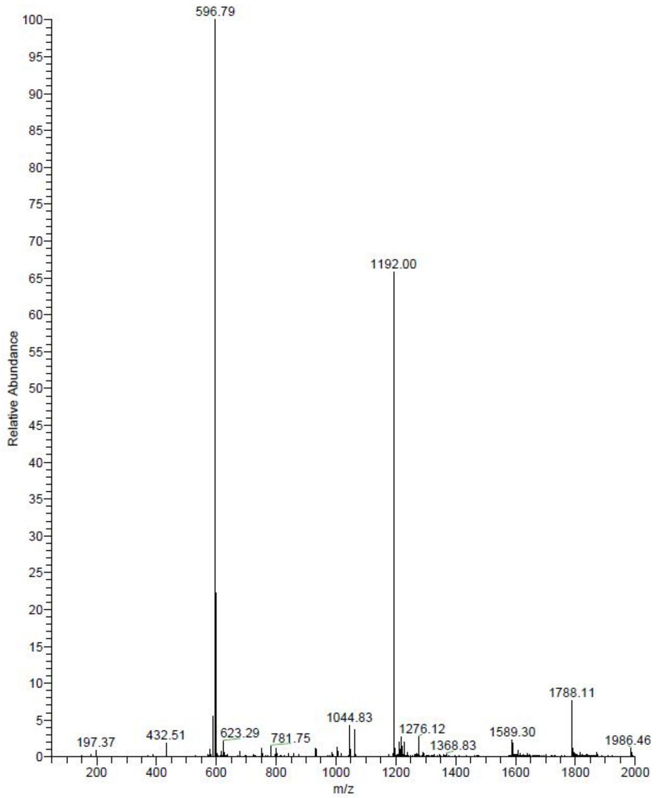

Supplementary Figure 22. Mass of H-YGFGL-NH-Succinic-YGFGL-NH_2 decapeptide. Calculated: 1190.58; found: 1192.00 $[M+H]^+$, 596.79 $[M+2H]^{2+}$

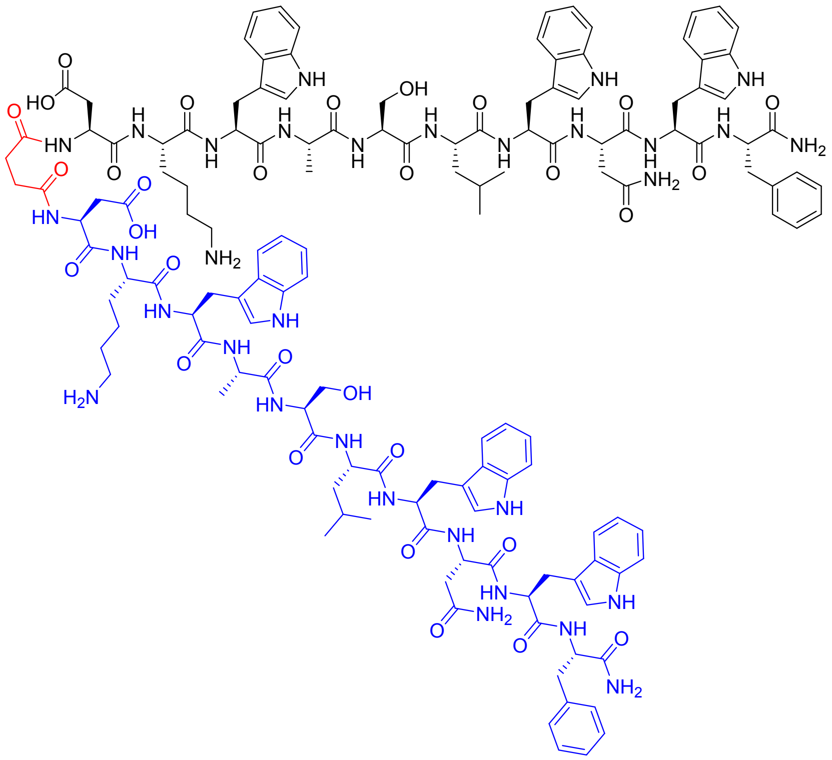

Chemical Formula: $C_{140}H_{172}N_{30}O_{32}$
Exact Mass: 2785.28

Supplementary Figure 23. Chemical structure of two-fragment (27-36) of Enfuvirtide (T-20 or Fuzeon) linked via succinamide, on Sieber amide resin. Black: first chain; red: succinic anhydride used to link the two chains; blue: second chain (PMR).

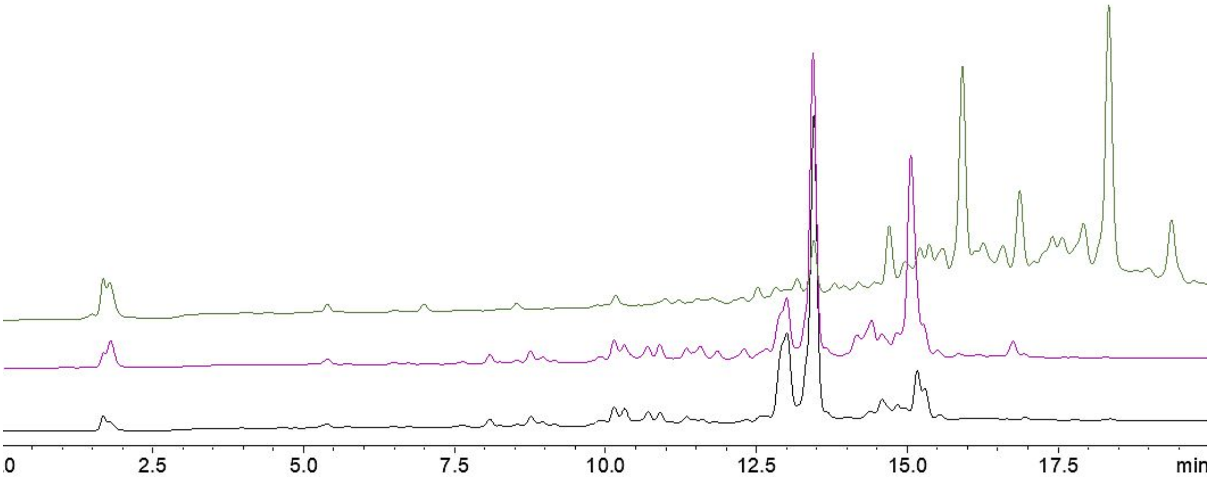

Supplementary Figure 24. Chromatograms of: black: one-fragment (27-36) of Enfuvirtide (T-20 or Fuzeon) (t_R= 13,5 min); pink: Succinic-T-20 (t_R= 15,1 min); green: two-fragment (27-36) of the same peptide (t_R= 18,4 min). 15–70% in 15 min gradient elution. Refer for the legend of Fig. S2 for the chromatographic conditions.

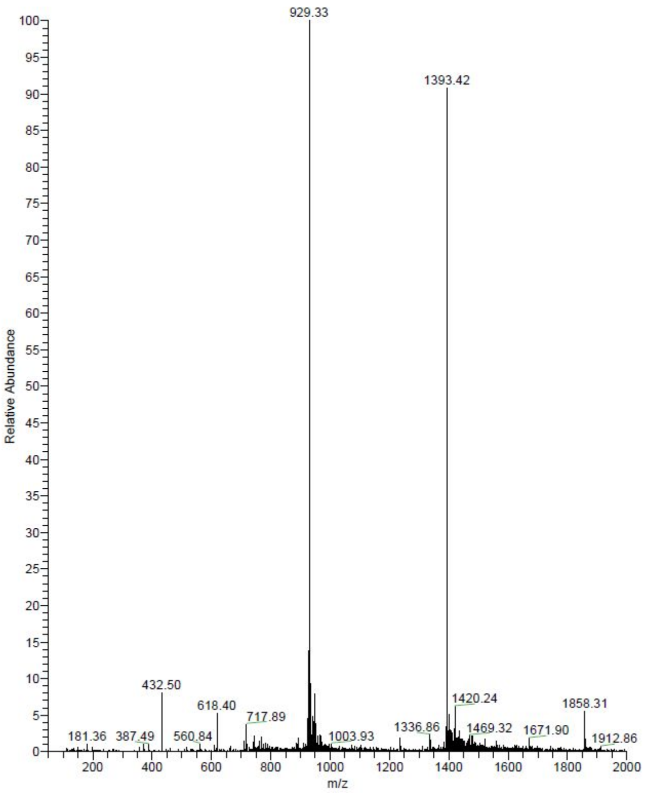

Supplementary Figure 25. Mass of PMR of T-20 fragment. Calculated: 2785.28; found: 1393.42 $[M+2H]^{2+}$, 929.33 $[M+3H]^{3+}$

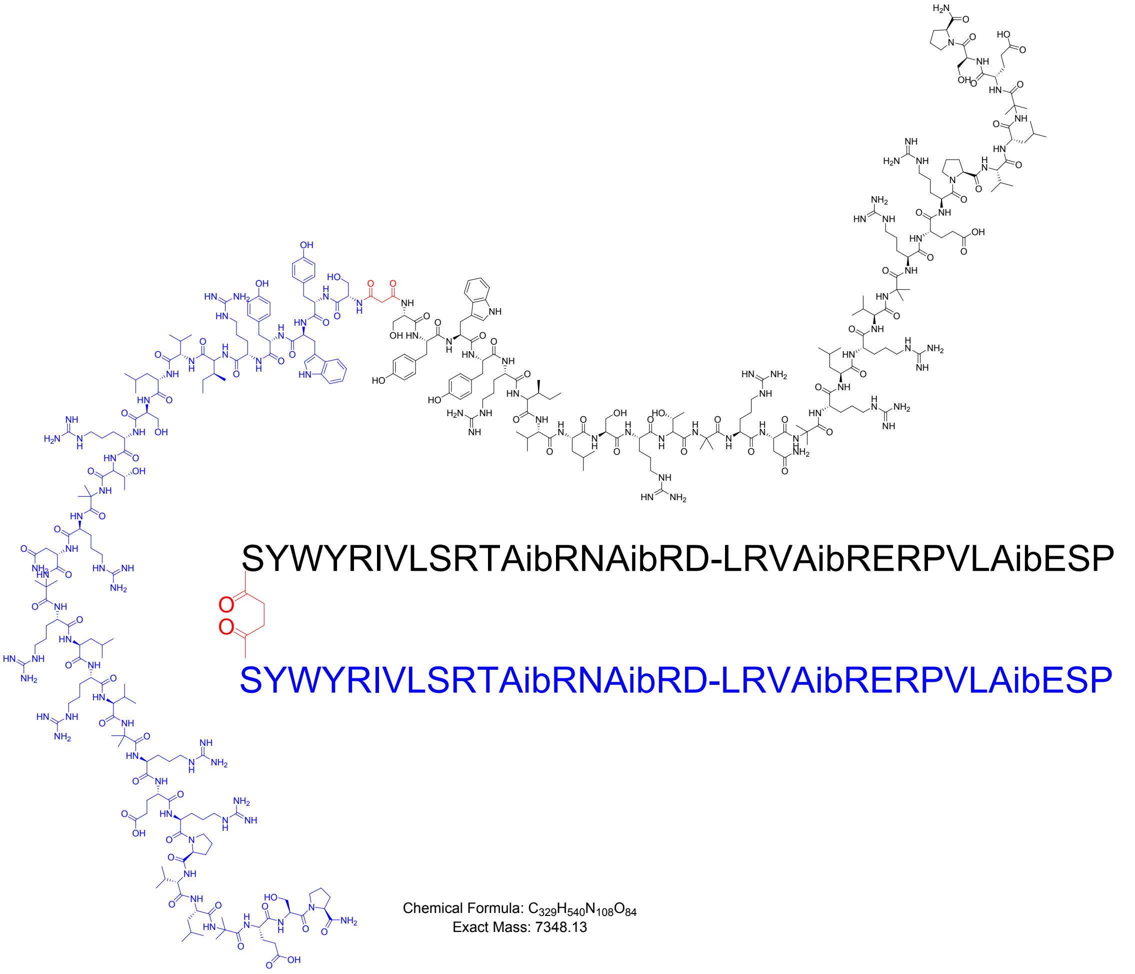

Supplementary Figure 26. Chemical structure of Aib modified TD2.2 peptide, two-fragment linked via succinamide, on protide resin. Black: first chain; red: succinic anhydride used to link the two chains; blue: second chain (PMR).

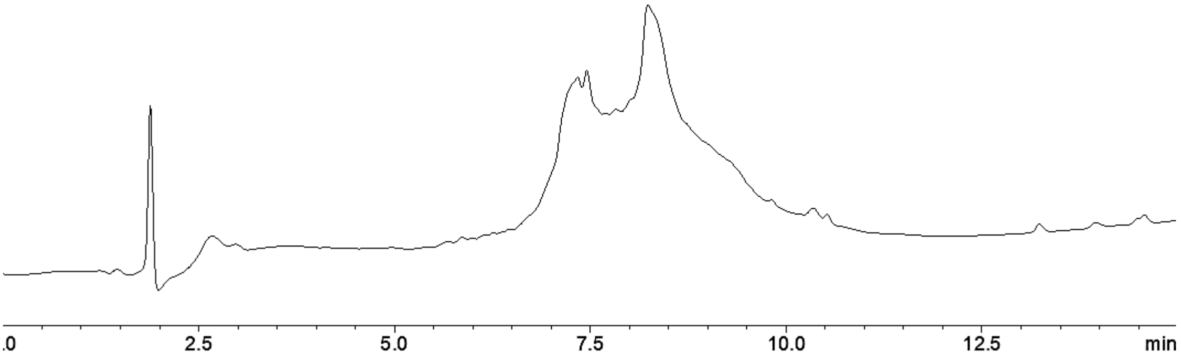

Supplementary Figure 27. Chromatograms of two-fragment of TD2.2-succinicamide-TD2.2 (t_R= 8.3 min). Refer for the legend of Fig. S2 for the chromatographic conditions.

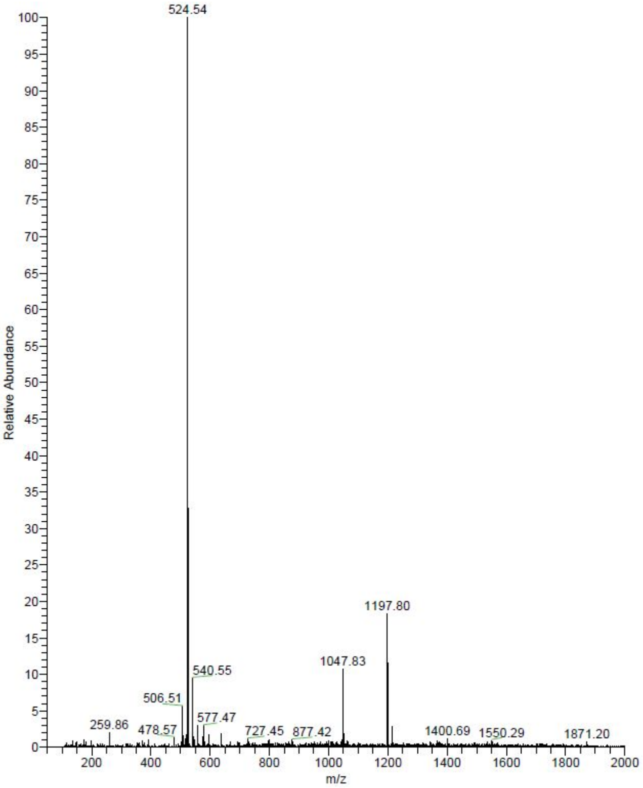

Supplementary Figure 28. Mass of PMR of modified TD2.2 fragment. Calculated: 7366.64; found: 1047.83 $[M+7H]^{7+\cdot}$, 524.54 $[M+14H]^{14+}$

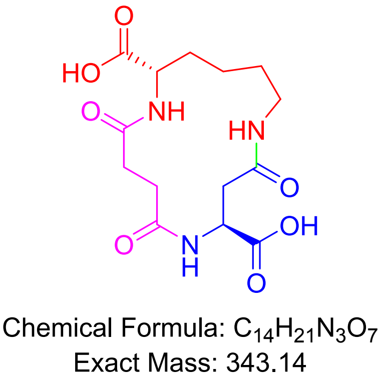

Supplementary Figure 29. Chemical structure of a new 2 amino acid cyclic peptide. Red: Lys; blue: Asp; pink: succinic amide; green: the formed peptide bond between sidechains.

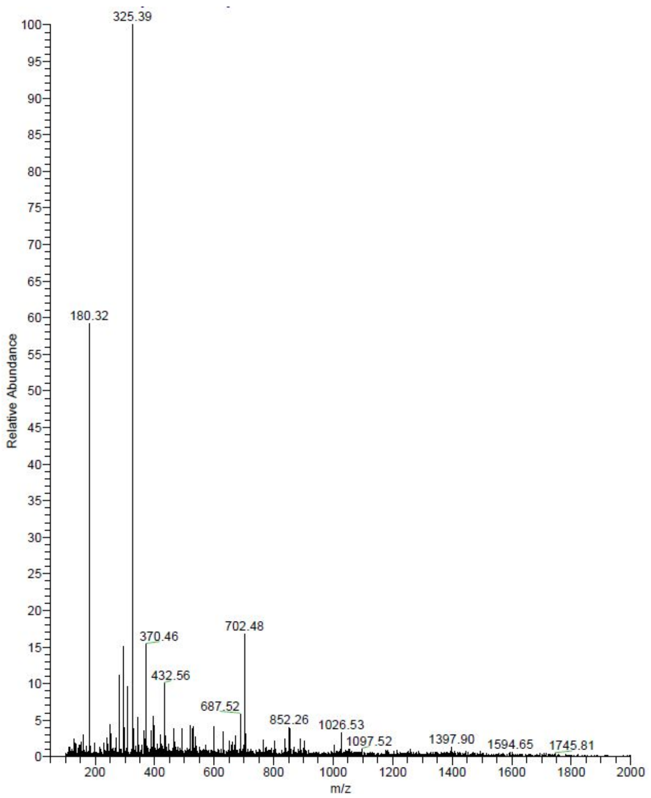

Supplementary Figure 30. Mass of cyclic dipeptide. Calculated: 343.14; found: 325.39 [M-18]$^+$

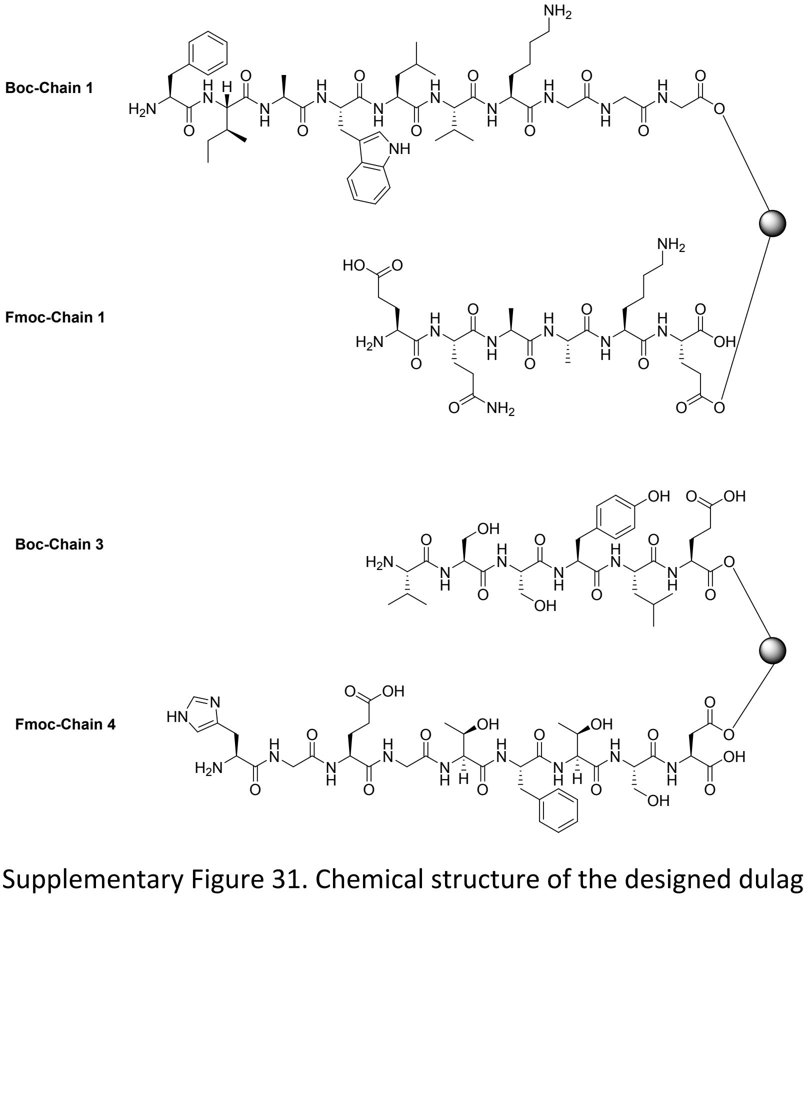

Supplementary Figure 31. Chemical structure of the designed dulaglutide fragments.

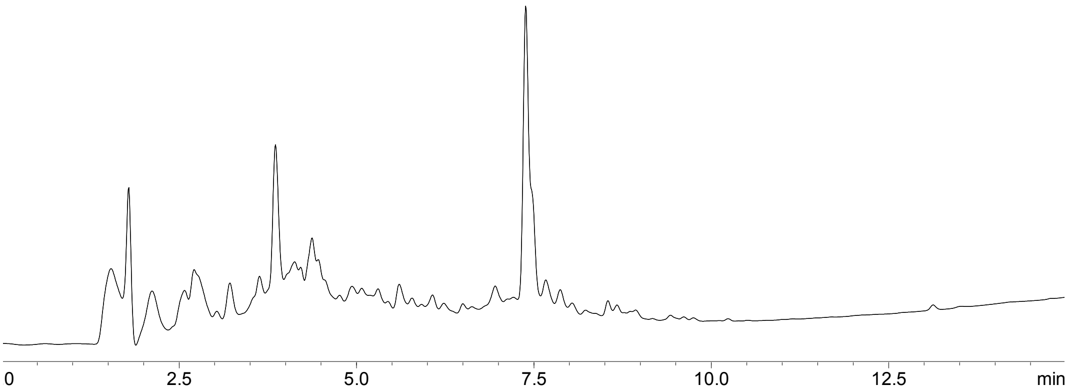

Supplementary Figure 32. Chromatograms of H-EQAAKEFIAWLVKGGG-OH (t_R= 7.5 min). Refer for the legend of Fig. S2 for the chromatographic conditions.

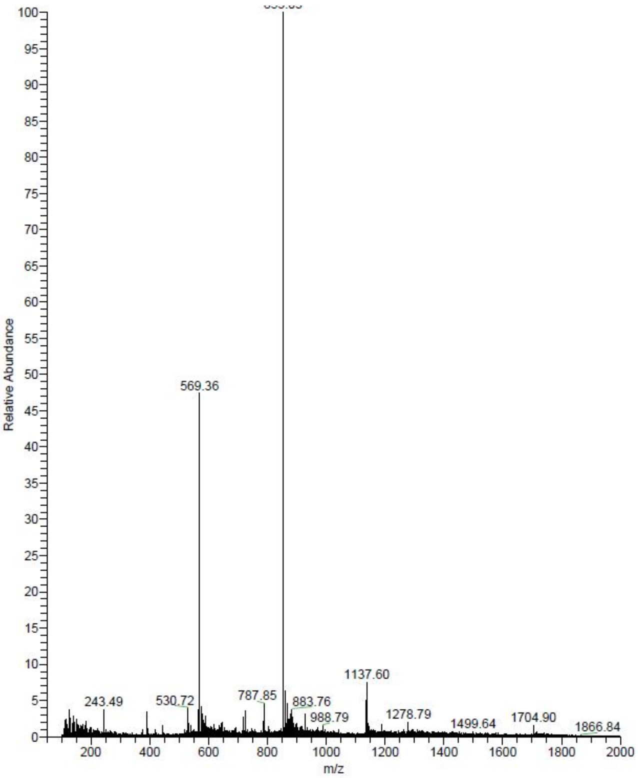

Supplementary Figure 33. Mass of H-EQAAKEFIAWLVKGGG-OH peptide. Calculated: 1703.96; found: 853.03 $[M+2]^{2+}$, and 569.36 $[M+3]^{3+}$

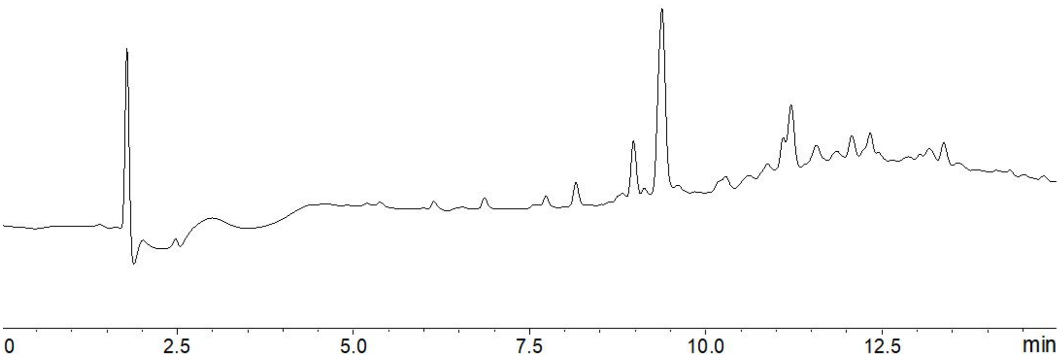

Supplementary Figure 34. Chromatograms of the triple cross-linked peptide (t_R= 9.4 min). 0−50% in 15 min gradient elution. Refer for the legend of Fig. S2 for the chromatographic conditions.

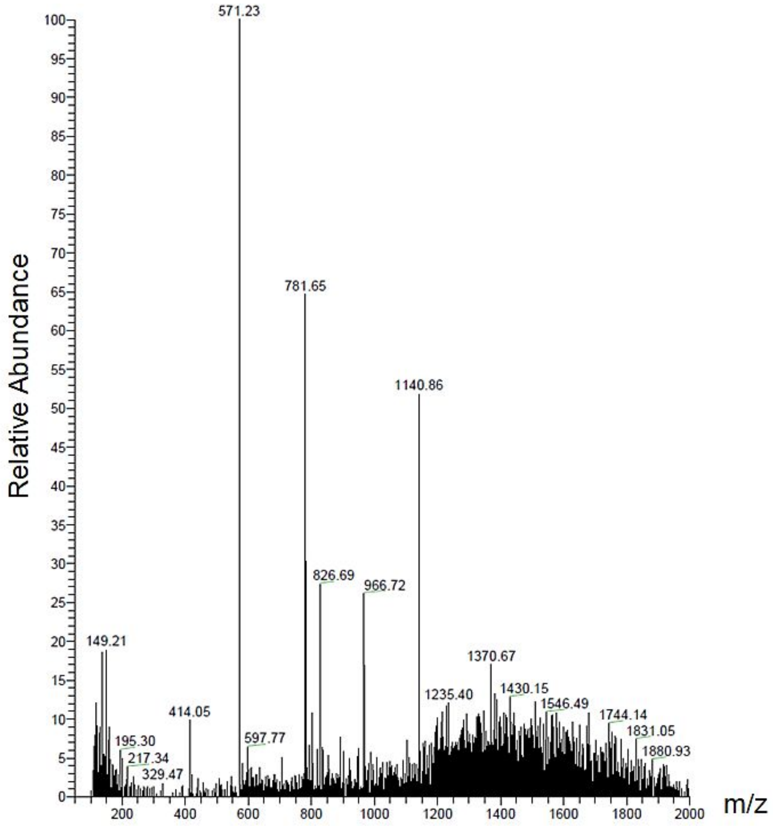

Supplementary Figure 35. Mass of crosslinked peptide. Calculated: 1140.26; found: 1140.86 $[M+H]^+$, and 571.26 $[M+2H]^{2+}$
